# Fra-1 regulates its target genes via binding to remote enhancers without exerting major control on chromatin architecture in triple negative breast cancers

**DOI:** 10.1093/nar/gkab053

**Published:** 2021-02-03

**Authors:** Fabienne Bejjani, Claire Tolza, Mathias Boulanger, Damien Downes, Raphaël Romero, Muhammad Ahmad Maqbool, Amal Zine El Aabidine, Jean-Christophe Andrau, Sophie Lebre, Laurent Brehelin, Hughes Parrinello, Marine Rohmer, Tony Kaoma, Laurent Vallar, Jim R Hughes, Kazem Zibara, Charles-Henri Lecellier, Marc Piechaczyk, Isabelle Jariel-Encontre

**Affiliations:** IGMM, Univ Montpellier, CNRS, Montpellier, France; PRASE, DSST, ER045, Lebanese University, Beirut, Lebanon; IGMM, Univ Montpellier, CNRS, Montpellier, France; IGMM, Univ Montpellier, CNRS, Montpellier, France; Medical Research Council, Molecular Haematology Unit, Weatherall Institute of Molecular Medicine, Oxford University, Oxford, UK; IMAG, Univ Montpellier, CNRS, Montpellier, France; LIRMM, Univ Montpellier, CNRS, Montpellier, France; IGMM, Univ Montpellier, CNRS, Montpellier, France; IGMM, Univ Montpellier, CNRS, Montpellier, France; IGMM, Univ Montpellier, CNRS, Montpellier, France; IMAG, Univ Montpellier, CNRS, Montpellier, France; LIRMM, Univ Montpellier, CNRS, Montpellier, France; Montpellier GenomiX, MGX, BioCampus Montpellier, CNRS, INSERM, Univ. Montpellier, F-34094 Montpellier, France; Montpellier GenomiX, MGX, BioCampus Montpellier, CNRS, INSERM, Univ. Montpellier, F-34094 Montpellier, France; Computational Biomedecine, Quantitative Biology Unit, Luxembourg Institute of Health, Strassen, Luxembourg; Proteome and Genome Research Unit, Department of Oncology, Luxembourg Institute of Health, Luxembourg, Luxembourg; Medical Research Council, Molecular Haematology Unit, Weatherall Institute of Molecular Medicine, Oxford University, Oxford, UK; PRASE, DSST, ER045, Lebanese University, Beirut, Lebanon; Biology Department, Faculty of Sciences-I, Lebanese University, Beirut, Lebanon; IGMM, Univ Montpellier, CNRS, Montpellier, France; LIRMM, Univ Montpellier, CNRS, Montpellier, France; IGMM, Univ Montpellier, CNRS, Montpellier, France; IGMM, Univ Montpellier, CNRS, Montpellier, France

## Abstract

The ubiquitous family of dimeric transcription factors AP-1 is made up of Fos and Jun family proteins. It has long been thought to operate principally at gene promoters and how it controls transcription is still ill-understood. The Fos family protein Fra-1 is overexpressed in triple negative breast cancers (TNBCs) where it contributes to tumor aggressiveness. To address its transcriptional actions in TNBCs, we combined transcriptomics, ChIP-seqs, machine learning and NG Capture-C. Additionally, we studied its Fos family kin Fra-2 also expressed in TNBCs, albeit much less. Consistently with their pleiotropic effects, Fra-1 and Fra-2 up- and downregulate individually, together or redundantly many genes associated with a wide range of biological processes. Target gene regulation is principally due to binding of Fra-1 and Fra-2 at regulatory elements located distantly from cognate promoters where Fra-1 modulates the recruitment of the transcriptional co-regulator p300/CBP and where differences in AP-1 variant motif recognition can underlie preferential Fra-1- or Fra-2 bindings. Our work also shows no major role for Fra-1 in chromatin architecture control at target gene loci, but suggests collaboration between Fra-1-bound and -unbound enhancers within chromatin hubs sometimes including promoters for other Fra-1-regulated genes. Our work impacts our view of AP-1.

## INTRODUCTION

AP-1 is a ubiquitous family of dimeric transcription factors (TF) that was identified >30 years ago (reviewed in (1)). In a restricted definition, it is defined as the collection of dimers made up of members of the Jun (c-Jun, JunB and JunD) and Fos (c-Fos, FosB, Fra-1/Fosl1 and Fra-2/Fosl2) multigene families. In contrast to the Jun family members, which can homo- or heterodimerize between them, the Fos family members must heterodimerize with one of the Jun proteins to bind to DNA. Fos:Jun dimers have a stronger affinity for DNA than the Jun:Jun ones and, usually, are also stronger transcriptional activators (see (1) and references therein). In an extended definition, AP-1 also includes the members of the ATF (ATF-2, ATF-3/LRF1, ATF-4, ATF-5, ATF-6B, ATF-7, BATF, BATF-2, BATF-3, JDP2) and MAF (c-MAF, MAFA, -B, -F, -G, -K and Nrl) multigene families (see (1)). The expression of the latter proteins is however more tissue/cell-specific than that of Fos and Jun proteins (2). Central in the biology of all of these proteins is the so-called bZIP domain made up of a basic domain allowing recognition of specific DNA motifs and an adjacent leucine zipper domain responsible for protein homo/heterodimerization. Depending on their composition, AP-1 dimers bind to specific types of palindromic sequences and their variants. Thus, Fos:Jun and Jun:Jun dimers preferentially bind DNA motifs referred to as 12-*O*-tetradecanoylphorbol-13-acetate (TPA)-responsive element (TRE; also called AP-1 motif), ATF-containing dimers preferentially bind to cAMP-responsive element (CRE) whereas MAF-containing dimers bind either MARE I or MARE II motifs that are extensions of TRE and CRE motifs, respectively (2).

A great wealth of information has accumulated on AP-1 regulation and physio-pathological functions, revealing that it is essential for virtually all cellular/physiological functions (3,4). Its components act as dynamic and versatile platforms to integrate many sorts of signaling events via different pathways (5–16) and are essential to translate these signals into transcriptome changes. Depending on the nature and duration of signals, as well as on the cell context, AP-1 component levels and activities can rapidly change to permit optimal responses to extracellular cues (3,4). Countless *in vitro* studies, as well as knockdown or overexpression of different AP-1 family members in mice, have highlighted the complexity of AP-1 biology. They have also indicated, at least for the Jun and Fos protein families, that their components can exert specific-, antagonistic-, as well as redundant actions, depending on the context (2).

Besides its physiological functions, AP-1 is also involved in diverse pathologies. The best-studied one is cancer where several of its components have been implicated in diverse tumorigenic processes (2,17–19). Oncogenic mutations of its component genes have rarely been reported. In fact, most protumoral effects of AP-1 are explained by its role as an essential effector of a variety of activated oncogenes. This is probably best-illustrated by the oncogenic events that can occur along the Ras pathway. These can be responsible for changes in the relative abundances of diverse AP-1-constituting proteins in tumor cells through transcriptional regulation of their genes and/or protein stabilization via alteration of post-translational modifications that can also affect transcriptional activity (1,2,5–7,19–25).

Despite its long-acknowledged transcription factor function, the gene targets of AP-1 and the fine molecular mechanisms through which it exerts its transcriptional actions still remain ill-characterized (1). An inherent difficulty for such studies is the large number of possible AP-1 dimers within the same cell, combined with the possibility of both antagonistic and redundant functions between these dimers. Moreover, before the advent of ‘omics’ studies, AP-1 molecular transcriptional studies were essentially focused on gene promoter regions, giving the biased impression that AP-1 principally regulates gene expression through binding close to, or at, gene promoters. However, this view has recently been challenged by genome-wide studies pointing to frequent binding of AP-1 at enhancers located far away from gene transcription start sites (TSSs), though the nature of its components was most often not established (1).

Fra-1 is the Fos family member that has most frequently been implicated in cancer. More specifically, it is overexpressed both at the mRNA and protein levels in many epithelial cancers in response to undue activation of various signaling cascades (5–7,14,15,25–30). There is substantial evidence that its overexpression contributes to tumorigenesis and tumor aggressiveness in pleiotropic manners that may differ according to the cancer type. These include promotion of cell division, resistance to apoptosis, inflammation, epithelial-mesenchymal transition, cell motility, tissue invasion and metastasis spreading (17,19,31,32). However, our knowledge on Fra-1 target genes and the mechanisms whereby Fra-1 regulates their transcription remains particularly limited. For example, it is still unclear whether Fra-1 impacts on chromatin conformation or whether it exploits preset 3D architecture networks to influence the expression of its target genes in the cancer cells where it is overexpressed. This question is important, as the recent literature suggests, not only that AP-1 may exert context-dependent transcription pioneering-, chromatin-remodeling- and chromatin accessibility maintenance actions, but also that certain AP-1-binding enhancers may be engaged in dynamic chromatin movements (see (1)). Yet, the specific AP-1 dimers at play were in general not characterized precisely in these studies.

To address this issue at the genome scale, we have turned here to Triple Negative Breast Cancers (TNBCs) for two main reasons. First, Fra-1 is overexpressed in most TNBCs where it plays a central part in their aggressiveness (17,19,31–36). Second, TNBC cells provide a relatively simple Fos family protein landscape facilitating the study of Fra-1, as they essentially, if not exclusively, express Fra-1 and Fra-2, with, however, much more Fra-1 than Fra-2 (37). As Fra-2, like Fra-1, can recognize the AP-1 transcription factor binding sites (TFBSs) and was reported to contribute to invasion in TNBCs (38), we also included it in our investigations. In addition to unveiling differences between Fra-1 and Fra-2, our work shows that Fra-1 principally controls its target genes via long distance interactions most probably implicating hubs engaging Fra-1-bound and Fra-1-non-bound regulatory elements without, however, affecting to any major extent chromatin architecture maintenance at target gene loci. Our observations have general consequences concerning the mechanisms whereby AP-1 controls gene expression.

## MATERIALS AND METHODS

### Cell culture

MDA-MB-231 cells were obtained from the American Type Culture Collection (ATCC, Rockville, MD, USA). They were cultured in Dulbecco's modified Eagle's medium containing 10% fetal calf serum and penicillin/streptomycin (100 μg/ml each) in a humidified 5% CO_2_ atmosphere at 37°C as previously described (37). Cells were routinely tested for the absence of Mycoplasma contamination.

### Antibodies

**Figure 1.**
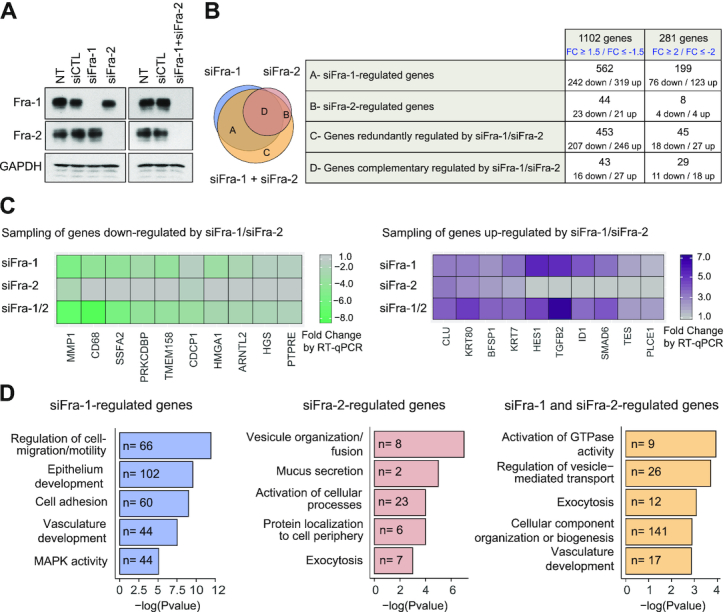
Transcriptomes controlled by Fra-1 and/or Fra-2 in MDA-MB-231 cells. (**A**) *Downregulation of Fra-1 and Fra-2 proteins upon siRNA transfection*. MDA-MB-231 cells were transfected with siCTL or siFra-1 and/or siFra-2 for 72 h. Cell extracts were analyzed by immunoblotting using antibodies specific for Fra-1 and Fra-2. GAPDH was taken as an invariant internal control. Left panels: single siRNA transfections. Right panels: double siRNA transfection. (**B**) *Transcriptomes regulated by siFra-1 and/or siFra-2*. siFra-1- and/or siFra-2-dependent transcriptomes were characterized using Affymetrix GeneChip Human genes 2.0ST array (see Supplementary Data S1A). The various groups of genes were defined as follows. For those preferentially regulated by siFra-1 (Group A), siFra-1 alone, but not siFra-2 alone, modulates transcript abundance. However, siFra-2 may strengthen the effect of siFra-1. For those regulated by siFra-2 (Group B), siFra-2 alone, but not siFra-1 alone, modulates transcript abundance. However, siFra-1 RNAi may strengthen the effect of siFra-2. For those redundantly regulated by siFra-1 and siFra-2 (Group C), siFra-2+siFra-1 modulates transcript abundance, whereas siFra-1 alone or siFra-2 alone do not. Finally, for those complementarily regulated by siFra-1 and siFra-2 (Group D), siFra-1 alone or siFra-2 alone modulates transcript abundance, but siFra-1+siFra-2 has stronger effects than individual siRNAs alone. Left panel: Venn diagram of genes regulated by siFra-1 and/or siFra-2. Right panel: numbers of up- and downregulated genes in each of the four classes of genes regulated by siFra-1 and/or siFra-2. The list of genes up- or downregulated by siFra-1 and/or siFra-2 is presented in Supplementary Table S1. (**C**) *RT-qPCR validation of siFra-1- and/or siFra-2-regulated genes*. A sampling of genes shown to be up- or downregulated by siFra-1 and/or siFra-2 in the Affymetrix array-based experiments was analyzed by RT-qPCR using RNAs prepared from MDA-MB-231 cells transfected as in A. Sequences of primers used for qPCR amplifications are given in Supplementary data S1B. (**D**) *Gene ontology analysis of genes regulated by siFra-1 and/or siFra-2*. Gene ontology analyses carried out using the GeneGo Metacore software are presented for the genes regulated by siFra-1, by siFra-2 or redundantly by siFra-1 and siFra-2 (FC ≥1.5 or ≤ −1.5). The top 5 informative non-redundant pathways in each category are listed along with their *P*-values and the number of regulated genes belonging to the pathway.

**Figure 2.**
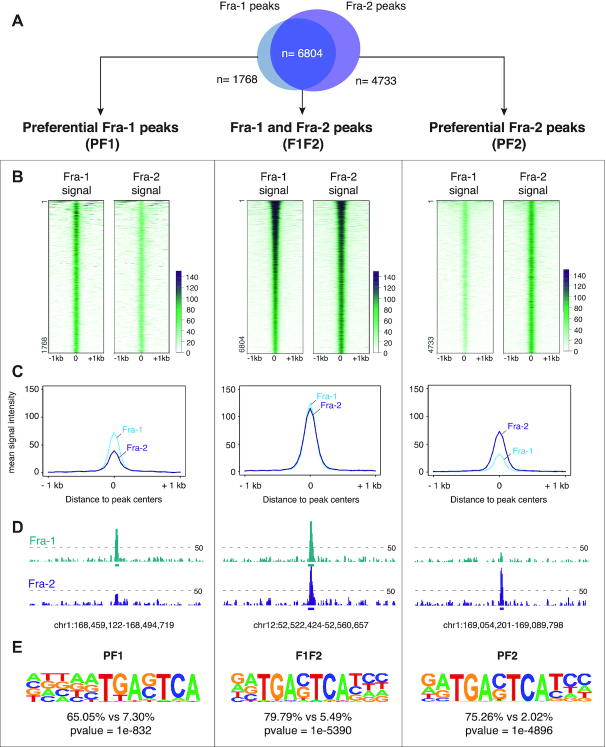
Fra-1- and Fra-2-binding sites in chromatin of MDA-MB-231 cells. MDA-MB-231 cells were cultured under standard conditions. (**A**) *Overlap between Fra-1 and Fra-2 ChIP-seq peaks*. Definitions of PF1, PF2 and F1F2 peaks are given in the text. (**B**) *Heatmap representation of Fra-1 and Fra-2 signals at* ±1 kb around PF1, F1F2 and PF2 peak centers. Regions were sorted according to Fra-1 decreasing signal intensity. (**C**) *Fra-1 and Fra-2 metaprofiles at ±1 kb around PF1, F1F2 and PF2 peak centers*. (**D**) *Examples of PF1, F1F2 and PF2 peaks*. The threshold for peak calling was set to 50 and is indicated by dotted lines (also see text). (**E**) De novo *motif analysis using HOMER*. The top-ranked motifs found in PF1, F1F2 and PF2 categories of sites are presented. Percentages indicate the fraction of peaks per group that contain the corresponding motif, as compared to a random set of genomic regions chosen as background.

The anti-Fra-1 (sc-376148X and sc-28310X), anti-Fra-2 (sc-13017X), anti-Pol II (sc-55492) and anti-GAPDH (sc-25778) antibodies were from Santa Cruz Biotechnology, as well as control IgGs (sc-2025). The anti-Fra-1 sc-376148X antibody was used in the ChIP-seq experiments of Figures 2 and 5A and immunoblotting assays of Figure 1A whereas a mix of sc-376148X + sc-28310X was used in ChIP-qPCR experiments presented in Figure 5C and Supplementary Data S7B. The anti-Fra-1 antibodies sc-376148X and sc-28310X recognize the N-terminus of Fra-1, which is not conserved in Fra-2. The anti-Fra-2 antibody sc-13017X was raised using as immunogen a domain encompassing residues [180–280], which is not conserved in Fra-1. The anti-Fra-2 antibody (D2F1E, #19967) used in ChIP-reChIP- and ChIP-qPCR experiments presented in Supplementary Data S7B and in Figure 5C, respectively, is from Cell Signaling. The latter experiments could not be conducted with sc-13017X, which was no longer available from Santa Cruz when we carried them out. The anti-Fra-2 antibody D2F1E is directed to the region surrounding Valine 245, which is not conserved in Fra-1. Immunoblotting experiments involving high loading of electrophoresis gels and overexposure of luminograms also formally ruled out the possibility of any cross-reactivity between the anti-Fra-1- and anti-Fra-2 antibodies used in this study. The absence of any cross-reactivity was also clear in the Re-ChIP presented in Supplementary Data S7B. The antibodies against H3K4me3 (Ab8580), H3K4me1 (Ab8895), H3K27ac (Ab4729) and p300/CBP (Ab14984) were from Abcam. The anti-p300/CBP antibody Ab14984 was used in ChIP-seq experiments conducted on MDA-MB-231 cells grown under standard cell culture conditions (Figure 5A), as well as in ChIP-qPCR experiments carried out on MDA-MB-231 cells transfected with either a control siRNA or siRNAs directed against Fra-1 or Fra-2 (Figure 5C, right panels). The p300/CBP antibody sc-32244X used in the ChIP-seq experiments conducted with MDA-MB-231 cells transfected with either a control siRNA or a siRNA directed against Fra-1 was form Santa-Cruz Biotechnology (Figure 5C, left panels, Supplementary Data S8C and D and Supplementary Table S3). The anti-CTCF antibody (C15410210) was from Diagenode.

### RNAi

For RNAi-mediated Fra-1 and/or Fra-2 depletion, we used pools of 3 siRNA directed to each protein (siFra-1 and siFra-2, respectively) and low siRNA concentrations to minimize potential off-target effects without altering on-target ones (see ref 37 for more details and for specific siRNA and control siRNA sequences or references). For simple Fra-1 or Fra-2 depletion, 4.5 nM (i.e. 1.5 nM of each siRNA constituting the pools) siFra-1 or siFra-2 were used in 72 h-long experiments. For depletion of both proteins together, 9 nM (4.5 nM of siFra-1 + 4.5 nM of siFra-2) of siFra-1+siFra-2 were used. siRNA transfections were conducted using INTERFERin (Polyplus) according to the supplier's specifications. For transcriptomic analysis (see Supplementary Data S1A), we included two control conditions: cells transfected with a control siRNA (siCTL, 4.5 nM for single depletion, and 9 nM for double knockdown) and cells placed in the presence of the transfection agent without siCTL (NT) to take into consideration possible off-targets effects of the control siRNA.

### Microarray-based whole-transcript expression analysis and profiling

RNAs were extracted using the Qiagen miRNeasy kit according to the supplier's specifications. On-column DNAse I digestions were performed as described in the user's guide. Three independent replicates were prepared for each condition (single transfections: NT, siCTL, siFra-1, siFra-2 and double transfections: NT, siCTL, siFra-1 + siFra-2) and controlled for purity and integrity using the Agilent 2100 Bioanalyser with RNA 6000 nano LabChipkits (Agilent technologies). Only RNAs with neither signs of contamination nor of degradation (RIN > 9) were further processed to generate amplified and biotinylated sense-strand cDNA targets using the GeneChip WT PLUS Reagent kit from Affymetrix according to the manufacturer's specifications. After fragmentation, cDNA targets were used to probe Affymetrix GeneChip Human Gene 2.0 ST arrays, which were then washed, stained and scanned according to Affymetrix instructions (user manual P/N 702731 Rev. 3). Importantly, RNA level changes were compared, not only to control cells transfected with equivalent amounts of control siRNA (siCTL), but also to cells that solely received the transfection reagent (NT) to eliminate false positive genes more stringently (also see Supplementary Data S1A)

### Microarrays, data analysis and gene ontology

A total of 21 CEL (seven conditions, *n* = 3 each) files generated after array scanning were imported into the Partek Genomics Suite 6.6 (Partek) for preprocessing consisting of estimating transcript cluster expression levels from raw probe signal intensities. Analyses were performed using default Partek settings. Resulting expression data were then imported into R (http://www.R-project.org/) for further analysis. First, nonspecific filtering was applied to remove transcript clusters with no specified chromosome location. Then, box plots, density plots, relative log expressions (RLEs), and sample pairwise correlations were generated to assess the quality of the data. They revealed no outlier within the series of hybridizations. Principal component analysis (PCA) was also applied to the data set. The first two components of the PCA were able to separate samples, except the non-transfected controls, according to the conditions. Thus, the siRNA transfection was considered as the unique source of variability. Finally, the LIMMA package (39) (R/Bioconductor) was used to detect differentially expressed genes between specific siRNA-transfected cells and control conditions. *P*-values were adjusted by Benjamin and Hochberg's false discovery rate (FDR) (40) and only genes with FDR <0.05 and modulated in the siRNA-specific conditions versus each associated control condition (siCTL and NT) were considered for further filtering, as described in Supplementary Data S1A. Gene Ontologies associated with the differentially expressed genes were obtained with GenGo (Metacore Software) and DAVID (https://david.ncifcrf.gov/summary.jsp).

### RNA extraction, reverse transcription and semi-quantitative PCR

RNA extraction, reverse transcription and semi-quantitative PCR were conducted as described in Tolza *et al.* (37). Sequences of primers used for PCR amplification are given in Supplementary Data S1B.

### Immunoblotting

Immunoblotting experiments were performed as previously described (41). Proteins were detected using the Luminata Forte Western HRP Substrate from Millipore.

### ChIP-seq

Chromatin immunoprecipitations (ChIPs) were performed as previously described (37,41) on MDA-MB-231 cells grown under standard cell culture conditions or on MDA-MB-231 cells transfected with either a control siRNA or siRNAs against Fra-1 or Fra-2, as indicated in the figures. Briefly, after 1% of paraformaldehyde (Euromedex) fixation at room temperature (24°C) for 5 min, cells were incubated in cell lysis buffer for 10 min. After mild centrifugation, nuclei were lysed in Nuclei Lysis Buffer (Tris–HCl 50 mM pH7.5, SDS 0.125%, EDTA 10 mM, NaButyrate 10 mM, protease inhibitors) at 4°C for 2 h and, then, sonicated for 10 cycles at 4°C using the Bioruptor Pico device from Diagenode. After sonication, chromatin absorbances at 280 nm (*A*_280_) of 1/100 diluted samples were measured and *A*_280 nm_-adjusted to 0.13 with the nuclei lysis buffer. For immunoprecipation of H3K4me1, H3K4me3 and H3K4me1, 150 μl of chromatin and 4.5 μl of the corresponding antibodies were used, whereas 400 μl of chromatin and 6.5 μl of antibody were used for CTCF immunoprecipitation and 600 μl of chromatin and 20 μg of the corresponding antibodies were used for that of Fra-1, Fra-2, Pol II and p300/CBP. Independent duplicates were prepared for all samples, and sequenced by the MGX genomic platform (Montpellier) using the Hi-seq2500 Illumina sequencer, except for the p300/CBP ChIPs where low amounts of chromatin were obtained and where the replicate samples were pooled before library preparation. p300/CBP ChIP-seqs conducted on MDA-MB-231 cells transfected with siCTL or siFra-1 were sequenced using the NovaSeq sequencer from Illumina. Data were aligned to the *Homo sapiens* Hg19 genome using the BWA-backtrack software, version 0.7.12–1039. Aligned reads were then processed using the R package PASHA (42) and the replicates were merged before peak calling. ChIP-seq peak calling was conducted using the thresholding function of the Integrated Genome Browser (IGB; https://bioviz.org). Thresholding parameters are presented in Supplementary Data S1C.

### ChIP-qPCR

Chromatin immunoprecipitation was carried out as for ChIP-seq. 100 μl of chromatin were used for Fra-1, Fra-2 and p300/CBP ChIPs, as well as in control experiments with irrelevant IgGs, using MDA-MB-231 cells transfected with either siCTL, siFra-1 or siFra-2. 3 μg of anti-Fra-1 antibody (1.5 μg of sc-376148X and 1.5 μg of sc-28310X), 10 μl of anti-Fra-2 antibody (D2F1E), 6 μl of anti-p300/CBP antibody (Ab14984) and 3 μg of control IgG (sc-2025) were used per ChIP. Immunoprecipitated chromatin was purified as described in Moquet-Torcy *et al.* (41) and Supplementary data S1F. Purified DNA was then subjected to qPCR analysis. The data were normalized with inputs taken from samples before the immunoprecipitation step and treated under the same conditions. Regions amplified and primers used for qPCR amplification are given in Supplementary Data S1H.

### NG Capture-C

The experiments were performed as previously described in (43). Material was obtained from MDA-MB-231 cells transfected with either siCTL or siFra-1 (4.5 nM siRNA for 72 h). Briefly, 3C libraries were generated using 900 U of the Dpn II restriction enzyme (NEB: R0543M) for 11 × 10^6^ cells and 720 U of the T4 DNA HC ligase (Life Technologies: EL0013). 3C libraries were sonicated (Bioruptor® Pico sonication device; 5 cycles; 30 s ON/OFF). Samples were indexed to allow multiplexing using Illumina paired-end sequencing adaptors (NEB: E6040, E7335) and the Herculase II polymerase (Agilent: 600677). Oligonucleotide capture was then performed using 70-mer biotinylated DNA oligonucleotides designed at each of the studied target gene promoters (http://apps.molbiol.ox.ac.uk/CaptureC/cgi-bin/CapSequm.cgi) (Supplementary Data S1D). The hybridization reaction was performed using Nimblegen SeqCap EZ kits (Roche: 05634261001, 07145594001, 06777287001). After a 72 h hybridization step, streptavidin bead pulldown (Invitrogen: 65305) was performed, followed by multiple washes (according to SeqCap EZ SR user's guide) followed by PCR amplification of the captured material. A second capture step was performed as above. The material was sequenced using the Illumina^®^ HiSeq 2500 platform with 125-bp paired-end reads. Data were analyzed using scripts available at https://github.com/telenius/captureC/releases and R was used to normalize data. Differential analysis between the siCTL and the siFra-1 condition was performed using DESeq2. Peak calling was performed using the PeakC R package (44) using the following parameters: alphaFDR = 0.1, wSize = 5 and qWr = 1. Adjacent restriction fragments called by PeakC were then merged into a single region using BEDTools (45).

### Motif analysis

Motif enrichment was analyzed using HOMER (http://homer.ucsd.edu/homer/ngs/peakMotifs.html). ChIP-seq peak sequences were compared to randomly selected genomic fragments of the average region size and matched for GC content (findMotifsGenome.pl mybed.bed hg19 output_directory -size given).

### TFcoop

To run TFcoop (46), we first identified all motif occurrences (PWM score ratio > 0.80) in each 1,001bp-long sequence using MotifSearch (option h 80) (https://hal-lirmm.ccsd.cnrs.fr/lirmm-01967466) and the maximum scores within each sequence were used as predicted variables. An equal number of sequences was considered for each class (Fra-1 and Fra-2 peaks). 70% and 30% of all sequences were considered for learning and testing, respectively. We used the seqLogo R package (Bembom, O. (2019) seqLogo Bioconductor, https://bioconductor.org/packages/seqLog) to reconstruct two PWMs from all occurrences of NFE2 with maximum scores in PF1 or PF2 peaks. To model the signal at F1F2 peaks, we first computed the log ratio log10(Y1/Y2) where Y1 and Y2 are the signals for Fra-1 and Fra-2, respectively. As for the classification, the maximal scores in each sequence calculated with all PWMs in the Jaspar database (662 PWMs) and all dinucleotide rates were used as predictive variables. For both classification and regression, a LASSO (Least Absolute Shrinkage and Selection Operator) regression was built using the cv.glmnet function from the glmnet R package, with 10-fold cross-validation and lambda.min.

## RESULTS

### Fra-1 and Fra-2 regulate either individually, together or redundantly genes associated with diverse biological processes in MDA-MB-231 cells

Human MDA-MB-231 cells have been employed in this study, as they constitute the most widely used and documented reference TNBC cell line model. Indeed, they show high fidelity with cells found in primary breast tumors with respect to, not only genomic and transcriptional aberrations (47), but also biological characteristics of tumor aggressiveness (dysregulated proliferation, mesenchymal-like phenotype, high migration activity, strong invasiveness and ability to form metastases in xenografted immunodeficient mice) (34,35,47,48). Moreover, MDA-MB-231 cells offer a relatively simple and favorable Fos family protein landscape to study Fra-1 transcriptional effects, as (i) they dramatically overexpress Fra-1, (ii) they also express Fra-2, albeit 15-fold less than Fra-1, and (iii) they show no detectable levels of Fos and FosB, as we previously reported (see Figure 2B and Supplementary Figure S1 of reference 37).

First, we identified the transcriptomes regulated by Fra-1 and/or Fra-2. This was the first transcriptomic comparison of these two TFs within the same cellular context. MDA-MB-231 cells were subjected to RNAi to downregulate Fra-1 and Fra-2 either individually or together and mRNA level variations were characterized using Affymetrix GeneChip Human Gene 2.0 ST arrays (see Materials and Methods and Supplementary Data S1A for details). Fra-1 and Fra-2 were efficiently depleted and downregulation of one protein did not affect the level of the other (Figure 1A). The transcriptomic data corresponding to 3 independent biological replicates were analyzed as described in Materials and Methods and Supplementary Data S1A.

The comparison of genes significantly regulated upon siFra-1, siFra-2 or siFra-1 + siFra-2 transfections identified four categories of genes (Figure 1B and Supplementary Table S1): those preferentially (up- or down-) regulated upon siFra-1 or siFra-2 transfection and those redundantly or complementarily regulated upon downregulation of Fra-1 and Fra-2 (see legend to Figure 1B for additional information). The expression of 1102 genes varied (positively or negatively) ≥1.5-fold with, among them, 281 varying ≥2-fold. Of note, most genes whose expression varied >2-fold belonged to the category of those regulated by Fra-1. Moreover, gene upregulation upon Fra-1 knockdown was slightly favored over downregulation, whatever the category of Fra-1-regulated genes (Figure 1B). RT-qPCR assays on a sampling of siFra-1- and/or siFra-2-modulated genes confirmed the transcriptomic results obtained using the Affymetrix technology (Figure 1C). Finally, gene ontology analyses using the GeneGo Metacore (Figure 1D) and DAVID (Supplementary Data S2) softwares indicated that the genes regulated upon the knockdown of Fra-1 and/or Fra-2 belong to a wide variety of functional categories. These analyses also pointed to a certain degree of specialization between the two proteins. On the one hand, the genes that were found principally regulated by Fra-1 were mainly involved in the control of cell migration and motility, which are the best described Fra-1-dependent pathological functions in TNBCs (32,34–36,48). On the other hand, the genes involved in vesicle organization and fusion were found mostly regulated by Fra-2, raising the possibility of a still undescribed Fra-2-dependent contribution to the TNBC phenotype.

### Common and specific binding sites for Fra-1 and Fra-2 in MDA-MB-231 cell chromatin

Next, we identified the genomic binding sites for Fra-1 and Fra-2 in MDA-MB-231 cells by ChIP-seq. This was achieved using non-cross-reacting antibodies recognizing unrelated epitopes in the Fra-1 and Fra-2 proteins (see Materials and Methods). Calling for enriched regions (see Materials and Methods and Supplementary Data S1C), 8572 peaks were mapped for Fra-1 and 11 537 for Fra-2. Their distribution analysis revealed a large overlap between Fra-1 and Fra-2 peaks with 79% of Fra-1 TFBSs also bound by Fra-2 and 59% of Fra-2 TFBSs also bound by Fra-1 (Figure 2A and Supplementary Table S2A). Though relative intensities of Fra-1 and Fra-2 signals may vary at a fraction of these overlapping peaks (Figure 2B, middle panel; see below for more information), their means were comparable (middle panel of Figure 2C for means and middle panel of Figure 2D for a typical example). Hereafter, these peaks are called ‘F1F2’ peaks for TFBS bound efficiently by both Fra-1 and Fra-2. Interestingly, when looking at peaks where only Fra-1 signals emerged above the threshold, much fainter Fra-2 signals between this threshold and the background noise were most often detected (left panels of Figure 2B, C and D for heat maps, means and typical example, respectively). Similarly, much fainter Fra-1 signals were found at most peaks where Fra-2 signals were detected above the threshold used (right panels of Figure 2B, C and D for heat maps, means and typical example, respectively). Hereafter, these peaks are called ‘PF1’ or ‘PF2’ peaks for TFBSs preferentially bound by Fra-1 or Fra-2, respectively. Note that Fra-1 and Fra-2 mean ChIP-seq signals at PF1 and PF2 peaks, respectively, were lower than that at F1F2 peaks (Figure 2B and C), suggesting lesser binding of the Fra proteins at these sites. Importantly, *de novo* motif analysis using the HOMER software revealed that the top-ranking motifs found in all three categories of sites (F1F2, PF1 and PF2) correspond to unambiguous TRE/AP-1 motifs (Figure 2E). This supported direct binding of Fra-1 and Fra-2 to DNA at the majority of observed TFBSs rather than indirect association via protein-protein interactions.

### Differences in sequence constraints for *in vivo* binding of Fra-1 and Fra-2 to AP-1 motifs

**Figure 3.**
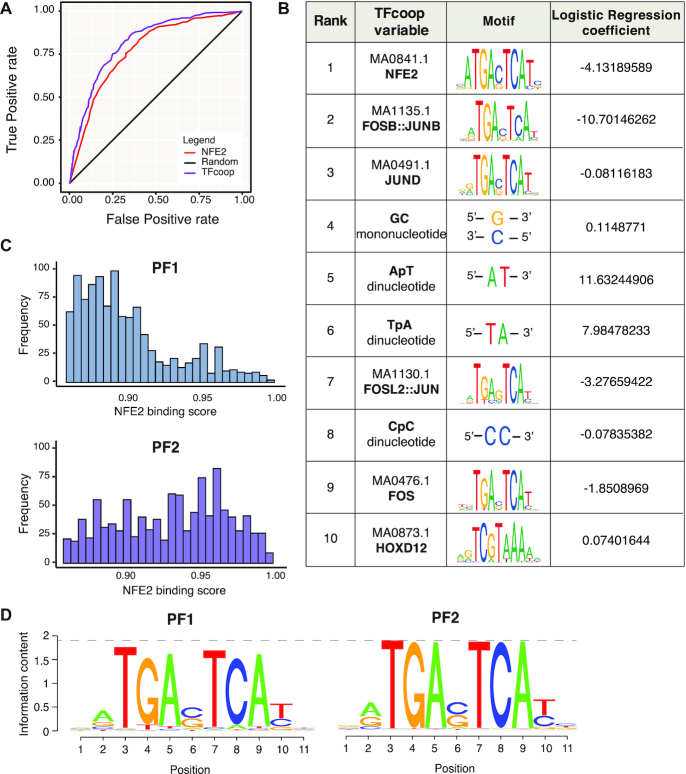
Binding preferences of Fra-1 and Fra-2 in MDA-MB-231 cells. (**A**) Classification of PF1 and PF2 TFBSs by TFcoop. The ROC curves show the accuracy of the PF1 and PF2 peak classification based on all parameters of TFcoop (purple) or using NFE2 PWM alone (red). Random classification is shown in black. (**B**) Top10 variables selected by TFcoop to classify PF1 versus PF2 peaks. The corresponding JASPAR PWMs and the logistic regression coefficients calculated by TFcoop are shown. The negative regression coefficients refer to the PF2 class, whereas the positive ones refer to the PF1 class. (**C**) Distribution of maximal NFE2 PWM scores (0.85–1) in PF1- (top panel) and PF2 peaks (bottom panel). (**D**) PWM reconstruction for PF1 and PF2 ChIP-seq peaks. The information content of binding sites on nucleotide sequences (83) describes how different the sequences are from all those possible in the genome of the organism, in a manner clearly delineating the important nucleotides of the site. Letter height in a sequence logo ranges from 0 bit (no base preference) to 2 bits (only one base used). Simply stated, the higher the letter corresponding to a nucleotide at a given position, the larger the information content and higher the probability of getting that nucleotide at that position. Overall, the heights of PF2 logo letters are higher than those of PF1 (see dotted line).

No overt difference in DNA-binding preferences between Fra-1 and Fra-2 has ever been reported thus far, whether *in vitro* or *in vivo*. However, the fact that we identified 3 categories of binding sites for Fra-1 and Fra-2 in our ChIP-seq analyses raised the possibility that these two proteins may, in fact, show some preference *in vivo*, at least at certain genomic sites. To address this point formally, we resorted to the TFcoop software (46), which predicts TF binding based on both the nucleotide content and the combinations of all JASPAR TF position weight matrices (PWM) scores computed in regions centered on ChIP-seq peak summits (1001 bp-long in the present analysis). This method has already allowed to improve the prediction of Fra-1- or Fra-2 binding to DNA in other cell types (46) without, however, comparing directly their binding preferences. We therefore asked whether TFcoop could also discriminate PF1 from PF2 peaks, i.e. would preferentially predict Fra-1-binding events to PF1 peaks and Fra-2-binding events to PF2 peaks. This turned out to be the case, as TFcoop classified PF1 and PF2 peaks with a good accuracy (AUC = 0.81, Figure 3A, purple curve), pointing to distinct binding preferences between Fra-1 and Fra-2 in PF1 and PF2 peaks *in vivo* in the vast majority of cases.

Then, we wished to get an insight into the molecular bases of Fra-1 and Fra-2 binding preferences. To this aim, we looked at the top10 variables selected by TFcoop for peak classification among the 662 variables used by this software (i.e. the 550 Jaspar PWMs + 2 mononucleotides + 10 dinucleotides). This revealed two interesting features. First, Fra-1 tends to bind regions richer in AT (variables 5 and 6 in Figure 3B), which is not the case for Fra-2. This conclusion was corroborated by analyzing dinucleotide distribution ±500 bp around Fra-1 or Fra-2 ChIP peak summits (see Supplementary Data S3), which showed higher AT content in the case of PF1 peaks. Second, among the 6 PWMs selected by TFcoop (Figure 3B), 4 corresponded to typical AP-1 TFs (variables 2, 3, 7 and 9) and 1 to an AP1-related TF (NFE2; variable 1). Importantly, NFE2 tolerates less sequence variations in the AP-1 core motif contained in the TFBS it recognizes than most AP-1 family TFs in the TFBS motifs they recognize (compare NFE2 and other AP-1 sequence motifs in Figure 3B). This suggested that the intrinsic nature of the AP-1 motif itself, rather than specific combinations with other transcription factors, may primarily specify the binding of the Fra proteins to DNA. Strengthening this idea, the classification of PF1 and PF2 peaks with TFcoop using only the first selected variable (i.e. NFE2 PWM) was nearly as accurate as that obtained when using all variables of TFcoop (Figure 3A; red curve; AUC = 0.75). This also implied that the variables not related to AP-1 motif sequences that were selected by TFcoop (e.g. dinucleotides and the HOXD12 PWM) contributed only moderately to discrimination between PF1 and PF2. The distributions of NFE2 PWM scores (see Materials and Methods) at PF1 and PF2 peaks were indeed significantly distinct, with high scores more frequently associating with Fra-2 peaks and lower scores with Fra-1 peaks (Figure 3C). This indicated that Fra-2 preferentially binds to sequences that are close to the canonical AP-1 motif encoded in the PWMs (high scores) while Fra-1 can bind more frequently sequences with some degenerate positions (lower scores). Reconstructing PWMs from PF1 and PF2 peaks confirmed this notion with the Fra-2 PWM exhibiting an information content higher than that of Fra-1 (see Figure 3D and its legend). Moreover, the canonical core AP-1 motif was found enriched with a log *P*-value of −2.825e+03 when searching for TFBS motifs in PF2 peaks with PF1 peaks as background (Supplementary Data S4A). However, this was not obtained in the reverse analysis considering PF1 peaks as foreground and PF2 peaks as background (Supplementary Data S4B). Thus, altogether, these observations indicated that Fra-2 binds more frequently than Fra-1 to DNA motifs most similar to the AP-1/TRE consensus sequence (TGAG/CTCA).

Interestingly, even though the vast majority of Fra-1 and Fra-2 signals detected at F1F2 peaks were comparable (middle panels of Figure 2B), differences in signal intensities were observed at a fraction of them (see Supplementary Data S5), raising the possibility that binding preferences applying to PF1 and PF2 peaks may also apply to certain F1F2 peaks. To test it, we modified TFcoop (see Materials and Methods), allowing it to perform a regression instead of a mere classification to predict the ratio between Fra-1 and Fra-2 ChIP signals at F1F2 peaks. Even though the correlation was high between Fra-1 and Fra-2 signals (*r* = 0.84), TFcoop nevertheless unveiled limited, though notable, differences in Fra-1 and Fra-2 bindings at F1F2 sites (the correlation between the predicted and the observed ratios was 0.31 with a *P*-value of < 2.2e−16). Consistently with the variables selected in the PF1 and PF2 classification task (Figure 3B), the first four variables selected by TFcoop in this regression analysis were NFE2 and AP-1 PWMs as well as AT nucleotides (Supplementary Data S6). This indicated that these variables can also differentiate, albeit to a modest extent, Fra-1- from Fra-2 binding at a fraction of F1F2 ChIP-seq peaks.

Next, we addressed the mechanisms behind Fra-1 and Fra-2 binding at F1F2 peaks. As Fos family proteins cannot heterodimerize with each other, co-binding of Fra-1 and Fra-2 at the same TFBS could not explain F1F2 peaks. Moreover, the idea that F1F2 peaks could predominantly be due to simultaneous binding of Fra-1 and Fra-2 at more than 1 site within the same ChIP-seq peak was not supported by the observation that the number of NFE2 occurrences was similar (median of 1) in PF1, PF2 and F1F2 peaks (only scores >0.8 were considered to limit false positives) (Supplementary Data S7A). Therefore, to discriminate between the possibility of Fra-1 and Fra-2 binding turnover at the same F1F2 TFBSs and that of recruitment of another Fra protein at another site within F1F2 peaks via protein-protein interactions, we resorted to ChIP-reChIP assays. In these experiments, we both (i) ChIPped Fra-1 and then reChIPped Fra-1 or Fra-2 and (ii) ChIPped Fra-2 and then reChIPped Fra-1 or Fra-2. The Fra-1 reChIP on Fra-1 ChIP, as well as the Fra-2 reChIP on the Fra-2 ChIP, served as positive controls to establish that the antibodies and method used were efficient in reChIP. We also used two negative controls: irrelevant IgGs as classical negative ChIP controls and empty beads to exclude any carryover of antibodies from the first ChIP. Ten genomic loci were selected for qPCR analysis of immunoprecipitated chromatin. Three were negative control regions showing neither Fra-1- nor Fra-2 signal in our ChIP-seq experiments. The 7 others corresponded to F1F2 peaks located in cAEs showing high Fra-1- and Fra-2 signals to provide optimal conditions for detection of possible Fra-1 and Fra-2 co-binding. No significant enrichment for Fra-2 reChIP on Fra-1 ChIP and, vice-versa, for Fra-1 reChIP on Fra-2 ChIP was found (Supplementary Data S7B). This indicated that (i) Fra-1 and Fra-2 do not predominantly co-bind at F1F2 peaks at the same time and (ii) detecting both Fra-1 and Fra-2 at F1F2 sites is most likely due to the fact that ChIP-seqs were performed on cell populations with the F1F2 sites being bound by Fra-1 in a fraction of cells and by Fra-2 in another one.

Thus, altogether, our data provide evidence that, in MDA-MB-231 cells, Fra-1 and Fra-2 show DNA-binding preferences at certain genomic sites. The molecular bases of this preference lie primarily in (limited) sequence variations within AP-1 TFBSs and, to a lesser extent, in the AT-content of the concerned regions. Importantly, in their vast majority, F1F2 peaks reflect neither the presence of several AP-1 TFBSs bound by the two Fra proteins nor direct binding of one Fra protein at the AP-1 TFBS and indirect protein-protein-mediated binding of the other Fra at another location within these peaks. Rather, they reflect the heterogeneity of the cell population we used in our experiments with turnover of Fra-1- and Fra-2-containing AP-1 dimers at the unique AP-1 site lying in these peaks.

### Fra-1 and Fra-2 principally bind to candidate active enhancers and little at gene promoters

**Figure 4.**
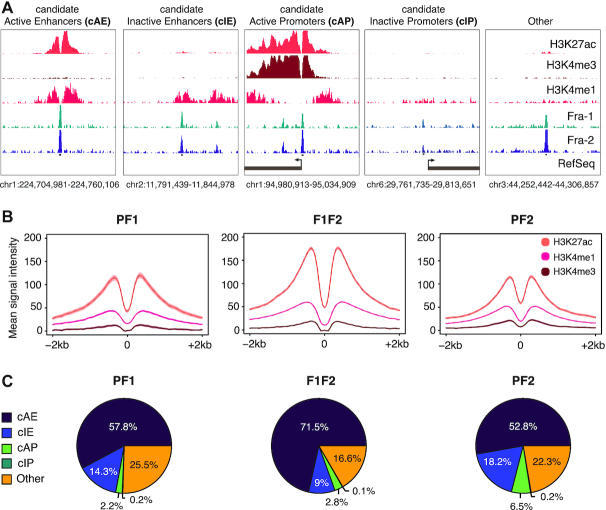
Fra-1 and Fra-2 principally bind to candidate active enhancers in MDA-MB-231 cells. (**A**) Annotation of candidate regulatory elements in MDA-MB-231 cells cultured under standard conditions. The level and distribution of H3K4me1-, H3K4me3- and H3K27ac marks, together with Ref-Seq promoter annotations, were used to define candidate active and inactive promoters and enhancers as explained in the text. (**B**) Histone mark metaprofiles at ±1 kb around PF1, PF2 and F1F2 peak centers. (**C**) Overlap of PF1, PF2 and F1F2 peaks with the different candidate regulatory elements defined in (A).

We next asked how Fra-1 and Fra-2 TFBSs were partitioned between enhancer and promoter elements. To this aim, we first mapped the latter regulatory elements in MDA-MB-231 cells. As there is neither a unique definition (whether functional, biochemical or operational), nor well-defined reporting guidelines for these elements (see Discussion and ref 50–53), we proceeded in several steps via resorting to a number of criteria classically used in the recent literature (also see next section). In the first step, using ChIP-seq, we mapped 3 histone modifications classically used to feature promoters and enhancers: H3K4me1 proposed to preferentially mark enhancers, H3K4me3 usually found in higher amounts at promoters than at enhancers and H3K27ac usually considered as a mark of activity for both promoters and enhancers (see ref 50–53). Based on this, we considered as (i) candidate active promoters (cAP), the 3 kb domains centered on RefSeq-annotated TSSs corresponding to protein-coding genes marked by H3K27ac, (ii) candidate inactive promoters (cIP), similar domains devoid of H3K27ac, (iii) candidate active enhancers (cAE), the regions marked by both H3K4me1 and H3K27ac but devoid of cAPs and cIPs, and (iv) candidate inactive enhancers (cIE), the regions marked by H3K4me1 but with low H3K27ac content and without cAPs and cIPs (Figure 4A). All genomic regions not corresponding to any of these categories were classified as ‘Other’. Using these criteria, 27,360 cAEs and 47,903 cIEs were identified (of which 26.5% and 2.3%, respectively, also showed H3K4me3 signals, albeit much fainter than at gene promoters). Among the 19 210 RefSeq-annotated promoters, 11,647 were cAPs (of which 97.5% were also marked by H3K4me3) and the rest (7563) were cIPs (of which only 6% were marked by H3K4me3). Importantly, we verified that the vast majority of cAEs (73.3%) and cAPs (88.6%) lay in open chromatin domains, as assayed by ATAC-seq in MDA-MB-231 cells (53), which was consistent with gene regulatory functions for them. In contrast, ATAC-seq peaks were found in only 25.4% of cIEs and 13.4% of cIPs, which suggested lesser, or no, regulatory activity for these elements (Supplementary Table S2B-E).

**Figure 5.**
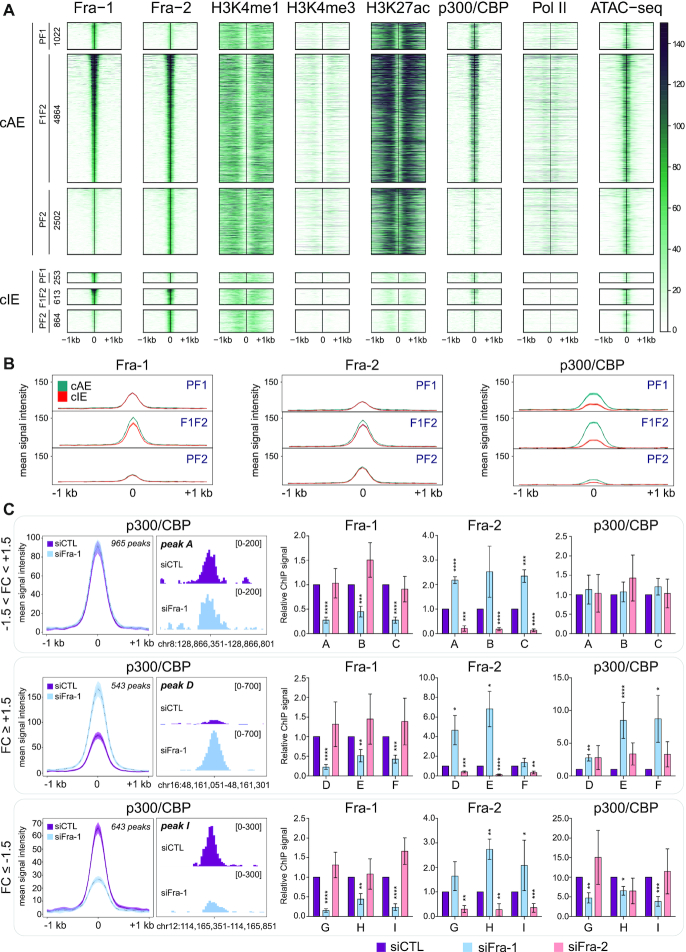
Epigenomic heterogeneity of candidate enhancers bound by Fra-1 and/or Fra-2. (**A**) Heatmaps of ATAC-seq (53) and ChIP-seq signals in MDA-MB-231 cells cultured under standard conditions at ±1 kb around PF1, F1F2 and PF2 peak centers at candidate active enhancers (cAEs; upper panel) and candidate inactive enhancers (cIEs; lower panel). Regions were sorted according to Fra-1 decreasing signal intensity and the number of regions in each category is indicated on the left. (**B**) Comparison of ChIP-seq metaprofiles for Fra-1, Fra-2 and p300/CBP at PF1, F1F2 and PF2 peaks between cAEs (green) and cIEs (orange). (**C**) p300/CBP recruitment at F1F2 peaks in cAEs upon Fra-1 siRNA-mediated knockdown. The left panels represent the ChIP-seq metaprofiles of p300/CBP at ±1.5 kb around F1F2 peak centers in cAEs of MDA-MB-231 cells transfected with either siCTL or siFra-1 for 72h. Regions were sorted according to the p300/CBP signal-fold change in siFra-1- versus siCTL condition. The threshold was set up to ±1.5, defining three types of regions: regions with −1.5 < FC < 1.5, FC ≥ 1.5 and FC ≤ −1.5. Middle panels represent screen captures of the ChIP-seq profiles of peaks representative of each category of regions using the IGV software. The screen captures correspond to peaks A, D and I, which were analyzed by ChIP-qPCR, as presented in the right panels. These ChIP-qPCR experiments were carried out on MDA-MB-231 cells transfected with either siCTL, siFra-1 or siFra-2 (right panels). Three regions per category were tested: A, B and C are regions with −1.5 < FC <1.5; D, E and F are regions with FC ≥ +1.5 and G, H and I are regions with FC ≤ −0.5. Signals were normalized to inputs and to the siCTL condition set to 1 for each amplicon. Results are the mean of five independent experiments. *P*-values were calculated using a two-tailed unpaired *t*-test (Prism5 software). (*), (**), (***) and (****) correspond to *P*-values of ≤0.05, ≤ 0.01, ≤ 0.005 and ≤ 0.0001, respectively. Coordinates of the regions analyzed and sequences of the primers used are given in Supplementary Data S1H.

In the second step, we intersected the above data with our Fra-1 and Fra-2 ChIP-seq data. Interestingly, the vast majority of Fra-1 (90.2%) and/or Fra-2 (90.5%) peaks fell into open chromatin domains (Supplementary Table S2A). Moreover, metaprofiles showed that H3K4me1-, H3K27ac- and H3K4me3 marks distributed bimodally around Fra-1 and/or Fra-2 peak centers (Figures 4B and 5B), which is typical of regulatory elements bound by transcription factors (54). Further analysis indicated that Fra-1 and/or Fra-2 proteins principally associated with candidate enhancers (72.1%, 71% and 80.5% of PF1, PF2 and F1F2 peaks, respectively) and much less frequently with promoters (2.4%, 6.7% and 2.9% of PF1, PF2 and F1F2 peaks, respectively) (Figure 4C and Supplementary Table S2F). Finally, and most interestingly, Fra-1- and Fra-2 were predominantly found in candidate active regulatory elements, whether promoters or enhancers (Figure 4C), this trend being stronger for F1F2 peaks in the latter case (Figure 4C and Supplementary Table S2F). Interestingly, candidate Fra-1- and/or Fra-2-bound enhancers were mapped distally from promoters, i.e. dozens to hundreds of kb from the closest promoters with a median distance of 50 kb.

Thus, taken together, our data strongly suggested that Fra-1 and Fra-2 bind preferentially to candidate active regulatory elements in MDA-MB-231 cells with a much stronger propensity to bind enhancers than promoters.

### Epigenomic heterogeneity of candidate enhancers bound by Fra-1 and Fra-2

We next characterized further the molecular features of Fra-1- and/or Fra-2-bound cAEs (8388 elements) and cIEs (1730 elements) (Supplementary Table S2F). The two highly related lysine acetyl transferases (KATs) p300 and CBP often associate with active enhancers marked by H3K27ac and RNA Polymerase II (Pol II) is responsible for bidirectional transcription of small, unstable, low abundance RNAs (eRNAs) at many enhancers (49–52). We therefore mapped p300/CBP and Pol II on the MDA-MB-231 genome and then addressed their presence at Fra-1 and/or Fra-2 TFBSs. We detected 9851 p300/CBP peaks (Supplementary Table S2A). Even though the majority of p300/CBP peaks (61.9%) fell into cAEs, 30% of them localized in regions with neither enhancer nor promoter features (Supplementary Table S2B–E), as seen by others in another setting (55), whereas a very minor fraction was found at cIEs and promoters (1.3% and 0.8%, respectively). Our Pol II ChIP-seq data showed 25,808 peaks (Supplementary Table S2A). Many of them (37.7%), which were also the strongest, corresponded to active gene promoters, whereas 22.3% were found at cAEs, 6.4% at cIEs and 1.4% at cIPs (Supplementary Table S2B-E and Supplementary Data S8), the rest being mainly distributed on gene bodies. Finally, we intersected p300/CBP- or Pol II peaks with PF1, PF2 and F1F2 peaks at cAEs and cIEs (Supplementary Table S2G-H). p300/CBP was found at only a fraction of PF1, PF2 and F1F2 peaks, whether located within cAEs (45%, 13.3% and 58.1%, respectively) or cIEs (13.4%, 4.3% and 20.9%, respectively). Pol II was also found distributed heterogeneously at PF1, PF2 and F1F2 peaks from cAEs (14.9%, 22.1% and 26.4%, respectively) and nearly absent at cIEs (0.4%, 1.3% and 2.3%, respectively) (Figure 5A and Supplementary Table S2G–H). Moreover, the elements bound by p300/CBP were not necessarily bound by Pol II and vice-versa. Thus, on their own, the analyses of p300/CBP and Pol II pointed to a first layer of molecular diversity at PF1, F1F2 and PF2 sites, both at cAEs and cIEs.

Next, more quantitative analysis of the intensity of Fra-1, Fra-2, histone modifications, p300/CBP and Pol II ChIP-seq- and ATAC-seq signals using heatmaps, metaprofiles and correlation analyses provided further comprehension of the above molecular diversity. As expected, the marks usually associated with enhancer activity (p300/CBP, Pol II, H3K27ac and ATAC-seq signals) were stronger at cAEs than at cIEs and this was also the case for H3K4me1 and H3K4me3 marks (Figure 5A and B and Supplementary Data S8B). However, pairwise comparisons of H3K4me1-, H3K4me3-, H3K27ac-, p300/CBP-, Pol II ChIP-seq- and ATAC-seq signal intensities showed limited to no correlation at all at the various PF1, PF2 and F1F2 sites, whether in cAEs or in cIEs (median Pearson correlation coefficient of 0.27 at cAEs and of 0.07 at cIEs), which supported the notion of epigenomic diversity of Fra-1- and/or Fra-2-bound candidate enhancers. Besides this, Fra-1 and Fra-2 average signal intensities were comparable at cAEs and cIEs within each PF1, PF2 and F1F2 peak category (Figure 5B), indicating that Fra-1 and/or Fra-2 signal intensity is not *per se* a good predictor of putative enhancer activity. Finally, p300/CBP mean signals at cAEs were the highest at F1F2 and PF1 peaks while very low at PF2 peaks (Figure 5B), which suggested that Fra-1, but not Fra-2, may facilitate p300/CBP recruitment (see below) as previously observed at specific loci (37,41,56).

Taken together, the above data pointed to marked molecular heterogeneity of candidate enhancers whatever the category of elements considered (cAEs, cIEs, as well as their PF1-, PF2- or F1F2 subcategories). Importantly, in addition to being more numerous, cAEs containing F1F2 peaks also displayed, on average, stronger marks of activity (ATAC-seq, H3K27ac and p300/CBP signals) than those containing PF1 and PF2 peaks.

### Fra-1 positively or negatively regulates p300/CBP recruitment at many F1F2 cAEs

Next, we addressed whether Fra-1 could regulate p300/CBP recruitment at F1F2 cAEs. To this aim, we first compared p300/CBP signals in ChIP-seq experiments performed on MDA-MB-231 cells transfected with siCTL to those in cells where Fra-1 was down-regulated by siFra-1. Seventy-six percent of the p300/CBP peaks formerly found at F1F2 cAEs in MDA-MB-231 cells grown under standard culture conditions (see above) were also detected in MDA-MB-231 cells transfected with the control siRNA, indicating robust p300/CBP binding sites (Supplementary Data S8C). RNAi-induced down-regulation of Fra-1 resulted in (i) a decrease of p300/CBP signals with a fold-change ≤−1.5 at 30% of the peaks, (ii) an increase of p300/CBP signals with a fold-change ≥1.5 at 25% of the peaks and no, or very moderate, changes at the remaining 45% of peaks (Figure 5C, Supplementary Data S8D and Supplementary Table S3).

To confirm that Fra-1 can actually impact p300/CBP recruitment at certain F1F2 cAEs and, at the same time, address whether Fra-2 could also affect this recruitment, we ChIP-qPCR-assayed p300/CBP levels at 3 types of F1F2 cAEs after RNAi-induced Fra-1- or -Fra-2 down-regulation, i.e. at sites where p300/CBP recruitment was either increased, decreased or not affected by siFra-1 in our ChIP-seq experiments. As expected, Fra-1 and Fra-2 signals were found strongly reduced at all tested peaks upon transfection of their respective siRNAs and, in all cases, we confirmed the effects of siFra-1 on p300/CBP signals seen in ChIP-seq experiments (Figure 5C, rights panels). In contrast, no significant change in p300/CBP signals was detected upon Fra-2 down-regulation (Figure 5C, right panels). Of note, p300/CBP ChIP-seq- and ChIP-qPCR experiments were carried out with two different anti-p300/CBP antibodies (see Materials and Methods) with similar outcomes, which strengthened our conclusions. Moreover, we observed that Fra-2 signals at F1F2 peaks were globally increased upon RNAi-induced Fra-1 down-regulation, whereas those of Fra-1 remained unchanged upon Fra-2 down-regulation (Figure 5C, rights panels). The simplest explanation to these observations takes into consideration (i) the broad difference in Fra-1 and Fra-2 protein amounts in MDA-MB-231 cells, (ii) the ensuing differential probability for them to occupy F1F2 sites at a given time and (iii) the fact that experiments were conducted on cell populations. Fra-1 being much more abundant than Fra-2, it likely occupies many more F1F2 sites and, when down-regulated, the probability of liberated F1F2 sites to be occupied by Fra-2 is high, leading to increased signals. In contrast, due to its lower abundance, Fra-2 occupies less F1F2 sites at a given time and, when down-regulated, the number of new sites that Fra-1 can occupy is limited, leading to no detectable change in signals.

Thus, our data support a role for Fra-1 in p300/CBP recruitment at approximately 55% of F1F2 cAEs. This role is, however, not univocal, as Fra-1 down-regulation impacts positively or negatively the presence of p300/CBP, depending on the site considered. In contrast, our data do not allow to detect any major role for Fra-2 in the recruitment of p300/CBP in MDA-MB-231cells, at least at the tested sites.

### Promoters of Fra-1-regulated genes interact with multiple distant Fra-1-bound- and non-Fra-1-bound enhancers

**Figure 6.**
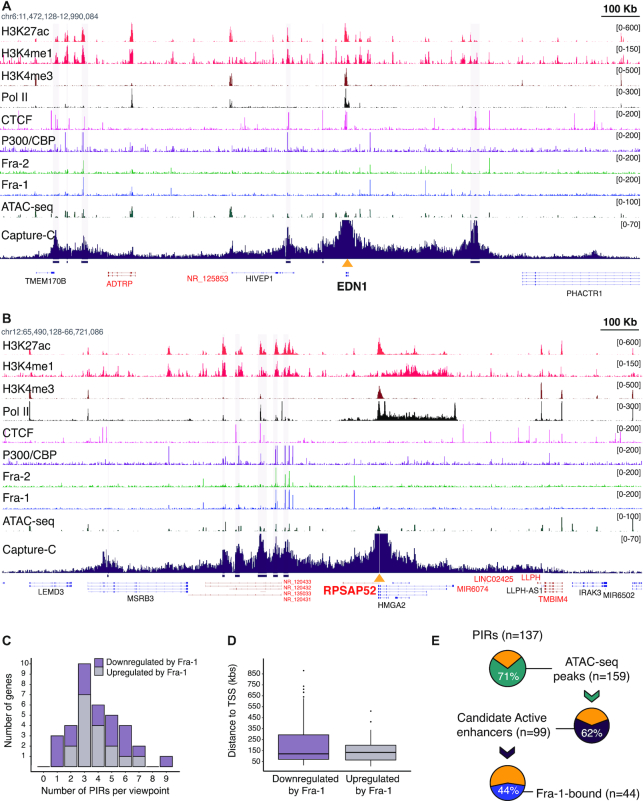
NG Capture-C analysis of Fra-1-regulated genes. (**A** and **B**) NG Capture-C profiles obtained at EDN1 (A) and RPSAP52 (B) gene loci. EDN1 is upregulated by siFra-1 whereas RPSAP52 is downregulated. The figures combine ChIP-seq, ATAC-seq and NG Capture-C data as indicated on the left. For NG Capture-C data on MDA-MB-231 cells transfected for 72 h with siCTL (see Figure 7B), the y-axis represents the normalized number of unique interactions per restriction fragment. PIRs identified using the PeakC R package are highlighted in light purple vertical lines. The scale (100 kb) is indicated at the top right edge of each figure. The yellow triangles indicate viewpoints. For better clarity, signals were strongly truncated at viewpoints. Genes encoded by the forward strand are shown in black and those encoded by the reverse strand are shown in red. (**C**) Distribution of PIR Numbers for the Fra-1 up- and downregulated genes. (**D**) Median distance between PIRs and viewpoints for Fra-1-up- and -downregulated genes. (**E**) Association of PIRs, candidate active enhancers and Fra-1. The fraction of PIRs containing ATAC-seq peaks is shown in green. The fraction of ATAC-seq peaks in PIRs with active enhancer marks is shown in purple. The fraction of the latter bound by Fra-1 is shown in blue.

To get insights on the regulation of Fra-regulated genes in MDA-MB-231 cells, we next aimed at identifying and characterizing the enhancers interacting with their promoters. We focused on Fra-1-regulated genes, as they are more numerous and were shown to have stronger expression changes in RNAi experiments than those controlled by Fra-2 (Figure 1). We opted for the NG Capture-C technique (43), which quantifies the chromatin interactions of selected multiple viewpoints genome-wide in a single experiment and at high resolution. The promoters of 34 genes, either up- (16) or downregulated (18) by Fra-1 (Supplementary Table S4) were chosen as viewpoints. Among these genes, three were redundantly regulated by Fra-1 and Fra-2 (*EMP1, SMAD6* and *TES*), three were complementarily regulated by the two Fra proteins (*KRT80, HEG1* and *PDE2A*) and the others 28 were preferentially regulated by Fra-1 (Supplementary Table S4). Results of the three independent biological replicates of the NG Capture-C experiments were merged, as they were highly reproducible (average Pearson correlation coefficient of 0.96 for all studied loci; see examples in Supplementary Data S9). The merged data on *EDN1* (downregulated by Fra-1) and *RPSAP52* (upregulated by Fra-1) are presented in Figure 6A and B as typical gene locus examples (also see Supplementary Data S10).

Using the PeakC R package (44), we identified 137 promoter-interacting regions (PIRs). For all genes, the most frequent interactions occurred in *cis*, i.e. on the same chromosome, within relatively confined regions that, overall, overlapped with topologically-associating domains (TAD) defined by Hi-C in other cell contexts (see examples in Supplementary Data S11). The number of PIRs *per* viewpoint varied from 1 to 9 with a median of 4 interactions (Figure 6C and Supplementary Table S4). The median distance between PIRs and their cognate promoters was ∼150 kbs (Supplementary Table S4 and Figure 6D), with the most proximal PIR lying 16 kb from the gene TSS and the most distal one lying 979 kb away (Supplementary Table S4). Importantly, no strong difference in the numbers of PIRs per promoter or in the distances separating PIRs from their cognate promoters was seen between the analyzed Fra-1-up- and -downregulated genes (Figure 6C and D; Supplementary Table S4), indicating that these features are unlikely to be major determinants discriminating these two gene categories. PIRs could be located exclusively upstream- (7 genes), exclusively downstream- (10 genes) or on both sides (17 genes) of TSSs, whether genes were up- or downregulated. Moreover, the PIRs located downstream of TSSs could be intragenic or reside in intergenic regions. These different features underlined differences in overall 3D locus topologies across the studied genes. Further supporting this point, annotated coding- and non-coding genes could be found between certain viewpoint promoters and their cognate PIRs, including the PIRs most proximal to TSSs (see Figure 6A and B for examples).

As a first step to apprehend the role of PIRs in the expression of the Fra-1-regulated genes, we intersected PIR-, ChIP-seq- and ATAC-seq data. 97/137 of PIRs harbored ATAC-seq peaks. PIRs were heterogeneous in size and many of them (39/97) contained more than 1 ATAC-seq peak, with a total of 159 peaks. Among these 159 ATAC-seq peaks located within PIRs, 99 held histone marks of candidate active enhancers. This strongly suggested regulatory functions for the latter regions. In addition, 44% (44/99) of these open chromatin sites showing histone marks associated with transcription activity were bound by Fra-1 (Figure 6E). Moreover, the vast majority of Fra-1-bound open regions within PIRs were also bound by Fra-2, as well as by p300/CBP and/or Pol II. Interestingly, a large fraction of the promoters interacting with Fra-1-bound PIRs also interacted with non-Fra-1-bound PIRs presenting marks of candidate active enhancers (Figure 6E). Altogether, this suggested that Fra-1-bound- and Fra-1-unbound enhancers may combine their action to regulate gene transcription.

Supporting direct transcriptional regulation by Fra-1, 9 Fra-1-up- (*FOSL1*, *CDCP1, SSFA2, PITPNC1, RPSAP52, HK2, MYO10, SOX9*,*SRGAP1*) and 11 Fra-1-downregulated genes (*TGFB2, IL8, ID1, HES1, KRT80, SMAD6, EDN1, ABTB2, GALNT2, LRP10, TCF7L2*) had their promoter regions interacting with at least one Fra-1-bound PIR displaying active enhancer histone marks (Supplementary Table S4 and Figure 6A and B for examples). Among them, *FOSL1* (which encodes Fra-1) deserves a special mention, as it is known to be subjected to positive autoregulation in certain tumors where the Fra-1 protein is stabilized and transcriptionally activated due to multiple phosphorylations (5,6,14,25,28,30,57). Thus far, only one regulatory element located within the first intron (at +1.1 kb) has been described as being involved in this regulation. However, its close proximity with the promoter meant that it falls below the resolution of NG Capture-C and could not be visualized in our experiments. In contrast, we did identify a novel PIR containing a Fra-1-bound cAE 17 kb upstream of the *FOSL1* TSS (Supplementary Data S10A). Moreover, a third Fra-1-bound cAEs (also falling below NG Capture-C resolution) was found 2.8 kb upstream of the TSS. It is therefore possible that the 3 enhancer elements cooperate to stimulate *FOSL1* gene expression in MDA-MB-231 cells.

No clear interactions between viewpoints and distal Fra-1-bound PIRs showing active enhancer features were found for 14 other genes (MAP2K1, ZNF114, PDEA2, MXD1, EMP1, HMGA1, ASB1, LIPH, TSPAN2, HEG1, TES, TMC6, FNDC3B, TNS3). Among them, however, PDE2A and ZNF114 might be genuine transcriptional targets of Fra-1, as our ChIP-seq indicated binding of Fra-1 at a cAE located in the viewpoint-proximal region for each of them. Their situation is reminiscent to that of HMGA1 that we have recently shown to be directly upregulated by Fra-1 in MDA-MB-231 cells via an intronic Fra-1-bound enhancer located close to the promoter (37). Moreover, our NG Capture-C data showed no additional Fra-1-bound regulatory elements at this locus, strengthening the idea that Fra-1-dependent upregulation of HMGA1 occurs exclusively by the intronic enhancer (Supplementary Data S10B). In contrast, the regulation of the remaining 11 genes by Fra-1 is likely to be indirect, as our ChIP-seq indicated no Fra-1 binding, not only in the viewpoint-proximal regions, but also at distal PIRs (see Supplementary Data S10C for illustration).

Finally, the analysis of PIRs also revealed that some of them harbor gene promoters of two possible types. On the one hand, some of these promoters belonged to genes shown to be regulated by Fra-1 in our transcriptomic analysis (e.g. PIR number 3 of the *KRT80* gene, harboring the promoter of the *KRT81* gene; Supplementary Table S4), suggesting sharing of common regulatory elements by two Fra-1-regulated genes. On the other hand, the other promoters belonged to genes not regulated by Fra-1, but held marks of putative activity (H3K27ac), raising the possibility that they may behave as E-promoters, i.e. promoters with enhancer activity on other genes (58) (e.g. PIR number 1 of the *FOSL1* gene containing the *CFL1* and *MUS81* gene promoters; Supplementary Figure S10A and Table S4).

In summary, our data suggest that (i) a large fraction (24/35) of the genes we have studied are direct transcriptional targets of Fra-1 in MDA-MB-231 cells, (ii) the promoters of these genes can interact with a variable number of (very) distal enhancers showing marks of putative activity that may, or not, recruit Fra-1, (iii) their expression may result from the combined action of Fra-1-bound and non-Fra-1-bound enhancers and (iv) certain Fra-1-regulated genes may share common regulatory elements with (at least) another Fra-1-regulated gene located within the same TAD.

### Fra-1 depletion does not affect overall 3D chromatin structure at studied Fra-1-regulated genes

**Figure 7.**
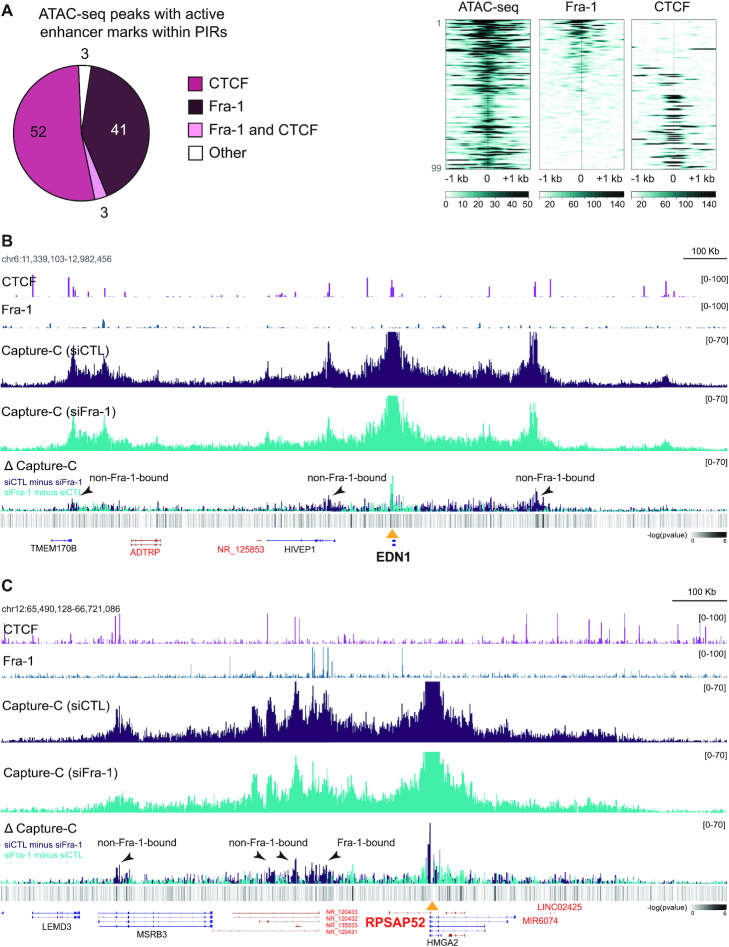
Limited effect of Fra-1 on overall 3D chromatin structure control at Fra-1-regulated gene loci. (**A**) Fra-1 and CTCF binding at PIRs with active enhancer marks and ATAC-seq peaks in MDA-MB-231 cells cultured under standard conditions. The left panel shows the distribution of the Fra-1 and CTCF ChIP-seq peaks and the right panel shows their heatmaps at the studied regions. For the heatmaps, the regions were sorted according to Fra-1 decreasing signal intensity. (**B** and **C**) Modulation of chromatin interactions by Fra-1 at the EDN1 (B) and RPSAP52 (C) loci. MDA-MB-231 cells were transfected for 72 h using siCTL or siFra-1 before NG Capture-C analysis. ΔCapture-C represents the differences in NG Capture-C signals between Fra-1-proficient (purple) and Fra-1-depleted (blue) conditions. CTCF and Fra-1 ChIP-seq data are presented above NG Capture-C data. DESeq2 analysis of the differential enrichment (minus log_10_ adjusted *P*-values) mapped across the loci is shown in the heatmap at the lower panels.

We finally addressed how long-range enhancer/promoter interactions of Fra-1-regulated genes could be controlled. Due to its major role in the control of chromatin architecture (59,60), we first addressed whether CTCF could be implicated in the formation/control of loops underlying the expression of Fra-1-regulated genes. To this aim, we mapped CTCF-binding sites in MDA-MB-231 cells by ChIP-seq. 39,059 peaks were identified (Supplementary Table S2A). We then focused on PIRs with active enhancer marks and centered them on ATAC-seq peaks (99 peaks). Our data showed that 55% (52+3/99) of these peaks overlapped with CTCF (Figure 7A), raising the possibility that CTCF may play a role in DNA looping between these PIRs and their cognate promoters. In contrast, 93% (41/44) of Fra-1-bound regions in PIRs with ATAC-seq peaks and active enhancer marks were devoid of any CTCF (Figure 7A), indicating a CTCF-independent mechanism for the formation of the chromatin interactions between, at least, these PIRs and their target promoters.

We next asked whether Fra-1 itself could control Fra-1-bound PIR/promoter interactions at the selected Fra-1-responsive genes. NG Capture-C profiles were compared between control- and Fra-1-depleted MDA-MB-231 cells where the loss of Fra-1 binding to PIRs was verified by ChIP-qPCR (Supplementary Data S12). Subtraction profiles of NG Capture-C data *(*Δ Capture-C) did not reveal any drastic global changes in NG Capture-C signals between the two conditions but revealed limited and subtle signal modulations at various places (see Figure 7B-C for examples). Taken together, these data suggested that, at least for the studied genes, Fra-1 is not required for maintenance of chromatin 3D structure and might exploit pre-established chromatin interactions between promoters and regulatory regions to exert its transcriptional actions. However, Fra-1 can exert limited modulatory effects on 3D chromatin architecture via modulating to a limited extent certain of these interactions. This was observed, not only at some Fra-1-bound PIRs, but also at some of those not bound by Fra-1, suggesting a possible communication between these PIRs.

## DISCUSSION

We report here that Fra-1- and Fra-2 RNAi-induced down-regulations modulate the expression of a large number of genes in the TNBC reference cell line MDA-MB-231 with, however, moderate effects on most of them. Resorting to RNAi was preferred over a gene KO-based approach to avoid selecting genes, the expression of which could indirectly be affected by long-term compensatory mechanisms linked to cell adaptation to Fra proteins depletion. Despite efficient down-regulation of both Fra-1 and Fra-2 in our experiments, we however do not exclude that, upon RNAi, trace amounts of these proteins may have marginally affected our data. Up- and downregulated genes were comparably abundant, suggesting that Fra-1 and Fra-2 may exert both positive and negative transcriptional effects in a gene-specific manner. Concerning Fra-1, this observation is consistent with that by Zhao et al. (36) who studied this TF in another TNBC cell line (BT549). Yet, further work is still required to establish extensively and unequivocally which, among all regulated genes, are genuine direct transcriptional targets of Fra-1 and Fra-2 transcription factors (also see below).

Fra-1 and/or Fra-2-regulated genes were found associated with a variety of biological processes, including cell motility, -migration and -adhesion, as well as angiogenesis and vesicular processes, which was coherent with the acknowledged pleiotropic effects of Fra-1 and Fra-2 in TNBC tumorigenesis and aggressiveness (17,19,31,61). As for Fra-1, our data were consistent with those by Zhao *et al.* (36) in BT549 cells where Fra-1-regulated genes were found distributed in functional categories largely overlapping ours. That we identified genes preferentially-, complementarily- or redundantly regulated by Fra-1 and/or Fra-2 supported the idea that the Fra proteins may regulate gene expression via diverse mechanisms utilizing them individually or in coordination (also see below). Indeed, the notion of diversity in the transcriptional actions of Fra-1 has begun to receive experimental support from the study of two genes it positively modulates in TNBCs (37,41). Importantly Fra-1, not only regulates many more genes than Fra-2 in MDA-MB-231 cells, but can also exert stronger transcriptional effects, as most of the genes varying by a >±2-fold factor were principally regulated by Fra-1 in our RNAi experiments. This is most probably due to Fra-1 higher abundance in TNBCs and is consistent with its more documented protumorigenic contributions in these tumors (17,19,31,61).

On the one hand, our Fra-1 and Fra-2 ChIP-seq experiments identified TFBSs preferentially bound by Fra-1 (PF1) or Fra-2 (PF2) that, most often, also show low but detectable ChIP-seq signals for the other Fra protein. On the other hand, they also identified TFBSs binding both Fra-1 and Fra-2 efficiently (F1F2) with, however, some differences in relative signal intensities between the two proteins. So far, no difference in DNA motif-binding preference has ever been reported between Fra-1 and Fra-2. However, our herein machine learning analyses revealed that the two Fra proteins can show differential binding efficacies, not only at PF1 and PF2 peaks, but also at F1F2 peaks, although in a less pronounced manner. The identifiable molecular bases of binding preferences lay primarily in (limited) sequence variations between AP-1 TFBS motifs, and, to a lesser extent, to the AT-content of the surrounding regions. Importantly, even though our findings reveal that TF’s preferred combinatorics do not distinguish Fra-1 and Fra-2 bindings in MDA-MB-231 cells, they do not rule out the possibility of cooperation between Fra-1 or Fra-2, on one side, and other TFs, on the other side (1,46). Moreover, we do not exclude that the more promiscuous behavior of Fra-1 may also be contributed by its overexpression in TNBCs, which would allow it to bind to TFBSs it would recognize less efficiently under (lower) physiological conditions of expression.

ChIP-seq- (Fra-1, Fra-2, histone modifications, Pol II and p300/CBP) and ATAC-seq data indicated that Fra-1 and Fra-2 bind unfrequently to promoter regions and very frequently to candidate active enhancers located distally from promoters. This strengthened the currently emerging notion that AP-1 would most often behave as a ‘remote command’ with a major role for long-range interactions in its transcriptional actions and not as a ‘local switch’ proximal to gene TSSs (1). Noteworthy, Fra-1- and/or Fra-2-bound cAEs displayed marked epigenomic heterogeneity. Thus, even though most sites showed association to open chromatin and active epigenetic marks, only a fraction was marked by p300/CBP and/or Pol II. Furthermore, the relative binding profile intensities for Fra-1, Fra-2, H3K4me1, H3K4me3, H3K27ac, p300/CBP and Pol II varied to a great extent from one to another site. We feel important to underline here the molecular diversity of the Fra-1- and/or Fra-2-bound regulatory elements for two reasons. First, the exact functional, operational, epigenomic and biochemical definition of enhancers is currently a matter of strong debate and there is currently accumulating evidence of their heterogeneity (see ref 50–53). Second, it raises the possibility of different mechanistic contributions to gene regulation for the various Fra-1- and/or Fra-2-bound candidate enhancers, including when they target the same promoter.

In this context, it is interesting to note that our RNAi/ChIP-seq experiments indicate that Fra-1 may have positive, negative or no effect on p300/CBP recruitment, depending on the F1F2 cAE considered. Along this line, fine analysis (Supplementary Table S4) of the F1F2 cAEs marked by p300/CBP at PIRs of the Fra-1-target genes we selected for our NG Capture-C experiments after treatment of MDA-MB-231 cells with siFra-1 pointed to another layer of complexity. In one case, we noted reduced p300/CBP signal at a siFra-1-down-regulated gene (Myo10), which was reminiscent of the positive correlation seen between, on the one hand, p300/CBP and, on the other hand, Fra-1 (41,56,62) and a number of other AP-1-constituting proteins (63–65) in transcriptional activity of various genes in other settings. Moreover, we noted increased p300/CBP signals at siFra-1-up-regulated genes (TGFB2, SMAD6, EDN1), which was also consistent with the common idea of positive transcriptional co-activator activity of p300/CBP. However, we observed no change in p300/CBP signals at certain siFra-1-up- (IL8 and GALNT2) or downregulated (SSFA2, RPSAP52 and ASB1) genes and reduced p300/CBP signal at a siFra1-upregulated gene (HES1). Several non-exclusive possibilities could be evoked to explain our observations. Since p300/CBP has been reported to interact, directly or indirectly, with many transcription factors (63–65), including Fra-1 (56) and other AP-1-constituting proteins (63–65), the final abundance of p300/CBP at a given enhancer might therefore reflect the balance between its positive and negative interactions with the different transcription factors binding to this enhancer, as proposed by others from studies in drosophila models (66). In this scenario, the post-translational modifications regulating transcription factor transactivation/repression activities might also be enhancer-specific and, thereby, key for regulating p300/CBP recruitment. In addition, p300/CBP activity being known to be regulated by cell signaling and post-translational modifications (63–65), one cannot exclude that p300/CBP activity has to be dissociated from its abundance at enhancers. Another possibility might lie in the fact that histone-modifying enzymes, including p300/CBP, are large proteins with multiple functional domains and may manifest non-histone-modifying activities that have been underestimated thus far (67). For example, scaffolding properties independent of lysine acetyltransferase activity have already been reported for p300 (68). The chemical tools directed to the different domains of p300 and CBP that are currently being developed may help addressing this question functionally (64).

To identify direct transcriptional targets of Fra-1 and their cognate regulatory elements, we conducted NG Capture-C experiments on 34 Fra-1-up- or -downregulated genes, taking their promoters as viewpoints. Most PIRs contained cAEs and most promoters interacted with multiple PIRs with a median of 4 interactions, i.e. a value consistent with the mean number of enhancers *per* gene proposed by others (1). PIRs were found distal from their cognate promoters with a median distance of 150 kb, albeit within the same TADs, underlining the role of long-range chromatin interactions in the regulation of the studied genes. There was no common locus configuration between the studied genes, as the numbers and locations of PIRs varied among genes. Moreover, genes of various sorts were seen between many studied promoters and their most proximal PIRs, strengthening the notion that assignment of enhancers to the nearest genes, although sometimes justified, is often not pertinent (49–52). Therefore, a number of the enhancer/promoter interactions proposed for Fra-1-regulated genes in BT549 cells on the basis of vicinity (36) probably have to be reconsidered. Among the 34 studied genes, 21 turned out to be highly likely direct transcriptional targets of Fra-1 since at least one of their PIRs (not necessarily being the most promoter proximal) was bound by Fra-1. Interestingly, the fact that Fra-1-bound- and non-Fra-1-bound PIRs bearing cAEs could interact with the same promoters suggested collaboration between them to control Fra-1-regulated gene transcription. Supporting this idea, recent work indicated that enhancer hubs can form owing to chromatin folding to allow for functional cooperation (70–78). Interestingly, certain PIRs of direct Fra-1 target genes were devoid of enhancer marks but carried promoters for other genes found regulated by Fra-1 in our transcriptome analysis. The network of physical and functional interactions at chromatin hubs might therefore not just be limited to one Fra-1-regulated gene promoter interacting with a limited number of PIRs but be more complex via including (at least) another Fra-1-regulated gene(s) and sharing of certain enhancers.

Fra-1-regulated genes were either up- or downregulated indicating that Fra-1 may act both as a transcriptional activator or a transcriptional repressor, depending on the gene context. This is an important notion concerning our view of Fra-1, as, after having initially been proposed to be a transcription repressor, Fra-1 was then considered as a transcriptional activator dependent on activating phosphorylations (6,20,21,24,25,79). It is also important to note that Fra-1-downregulated genes are expressed to notable levels in the presence of Fra-1. At this stage of investigation, it is however not clear whether this is due to the positive transcriptional action of non-Fra-1-bound cAEs, the effect of which would be limited by Fra-1-bound cAEs contacting them and/or their cognate promoters, or from those of regulatory elements contained within Fra-1-bound cAEs. The latter possibility would be consistent with the recent observation that many silencers and enhancers are embedded within the same bifunctional regulatory regions (80).

Deleting Fra-1 from MDA-MB-231 cells did not drastically alter chromatin interactions bringing about PIRs and promoters of Fra-1-regulated genes. This suggested that Fra-1 exerts its pleiotropic protumorigenic actions by principally exploiting the network of physical interactions and chromatin loop collisions occurring in these cells, instead of controlling global chromatin 3D organization. This observation raised, not only mechanistic-, but also biological questions from the tumorigenesis perspective, as, on the one hand, Fra-1 overexpression is not an initiator event in TNBCs and, on the other hand, we noted some differences in chromatin interactions at certain loci when comparing our PIR/promoter interaction data from MDA-MB-231 cells to high resolution Hi-C data obtained in non-tumorigenic primary human mammary epithelial cells (see Supplementary Data S10). Although we cannot exclude at this stage of investigations that such chromatin changes are consequent to Fra-1 protumorigenic activation, future work will have to elucidate which oncogenic event(s) establish(es) the particular chromatin 3D landscape that Fra-1 utilizes once it gets overexpressed. It will also be important to consider that these oncogenic events could also alter the level or activity of the transcription factors and co-factors Fra-1 collaborates with to exert its protumorigenic actions.

Our data raise the possibility that CTCF might be important in chromatin looping control at a number of non-Fra-1-bound PIRs. In contrast, CTCF is unlikely to be crucial for spatial positioning of Fra-1-bound PIRs, as it is rarely found at these sites. Along this line, it is interesting to note that, during macrophage differentiation, multi-loop activation hubs are established at key genes with enrichment of AP-1-bound enhancers in these hubs but with less frequent association of CTCF with AP-1-binding loops than with non-AP-1-binding ones (70). Nevertheless, deleting Fra-1 affected the frequency of certain long-range interactions to a limited but detectable extent. As these effects could be seen at both Fra-1-bound- and non-Fra-1-bound PIRs interacting with the same promoter, this pointed to possible communications between these two categories of PIRs and, indirectly, further supported the idea that enhancer hubs may actually form to control the transcription of a number of Fra-1-up and -downregulated genes.

Strikingly, the majority of TFBSs bound by one Fra protein was also bound by the other Fra, albeit to varying degrees, leading to the PF1, PF2 and F1F2 peak classification we suggested. Provided that (i) one single AP-1 motif is detectable at the vast majority of ChIP-seq peaks, (ii) the Fra proteins cannot homodimerize or heterodimerize between them and (iii) Fra-1 and Fra-2 ReChIP experiments conducted at certain F1F2 peaks argue against co-binding of Fra-1 and Fra-2 at the same sites, the most parsimonious explanation is that Fra-1- and Fra-2-containing AP-1 dimers turn over at these sites with our ChIP-seq data reflecting the average of Fra-binding events occurring in the population of analyzed cells. Moreover, we observed diverse transcriptional outputs (preferential, redundant or complementary up- or downregulation by Fra-1 and/or Fra-2) and different target gene configurations, in particular in numbers and positions of Fra-1- and/or Fra-2-binding PIRs, implying that the transcriptional actions of Fra-1 and Fra-2 at play are necessarily diverse. Taking this into account, different reasons for dynamic recruitment of Fra-1 and Fra-2 at the same TFBFs can be considered. For example, for genes complementarily regulated by the two Fra proteins but with only one PIR harboring a F1F2 peak, successive bindings of Fra-1- and Fra-2-containing AP-1 dimers might explain the complementary transcriptional effects of the two proteins, though the mechanistical reasons for AP-1 dimer turnover and differences in transcriptional outputs still need to be elucidated. For genes complementarily or redundantly regulated by the two Fra proteins with multiple PIRs harboring PF1-, PF2- and/or F1F2-containing cAEs, encounters of different PIRs at regulatory hubs would also provide a straightforward explanation. In contrast, the situation of genes preferentially regulated by Fra-1 that we analyzed by NG Capture-C is more puzzling, as most of the Fra protein-binding sites in the PIRs were of the F1F2 types. One possibility might be that only Fra-1 exerts a transcriptional action at these genes whereas Fra-2 remains neutral from a transcriptional standpoint for reasons that remain to be clarified. Alternatively, it cannot be ruled out that Fra-2 transcriptional potential is just masked by the much higher abundance of Fra-1. Whatever the explanation, future work will aim at clarifying these issues.

As Fos family proteins can bind to DNA only in the form of heterodimers (1), an important issue concerns the transcriptional partner, -or partners-, of Fra-1. Tackling this question, however, is particularly challenging for at least three main reasons. First, Fos proteins can, not only heterodimerize with all the members of the Jun family, but also with other ubiquitous bZIP transcription factors (1). Moreover, if we only take into account the Jun proteins, there is evidence that, even though they display clear specificities, they can also show partial functional redundancy (2). A second major reason lies in the observation that Fra-1 can exert both positive and negative transcriptional actions depending on its target genes, as illustrated here and elsewhere (19), which possibly engage different heterodimerization partner(s). Finally, it is also possible that the identification of the pertinent heterodimerization partners of Fra-1 may be complicated by the dynamic behavior of AP-1 as (i) our work strongly suggests a turnover of Fra-1- and Fra-2-containing AP-1 dimers at F1F2 sites and (ii) AP-1 dimers constantly form and dissociate in living cells with interaction times of less than a few minutes (81). Whatever the case and without underestimating the contribution of its heterodimerization partners, it is highly likely that Fra-1 plays a preponderant part in the transcriptional effects we report here, as Fos proteins have long been known to both display stronger transcriptional- and promoter-selection activity than their Jun partners in Fos:Jun heterodimers (82).

In conclusion, we have investigated here how Fra-1 regulates its target genes in a TNBC context due to its crucial role in the aggressiveness of this tumor type. Consistently with its pleiotropic tumorigenic effects, it appeared to modulate positively and negatively a wide array of genes with however moderate effects on most of them. In a number of cases, this is achieved in coordination with Fra-2, which is the other Fos family member expressed in these tumors albeit at a much lower degree and involves, at least at certain sites, differences in AP-1 motif recognition by Fra-1 and Fra-2. Our data also strongly suggest that Fra-1 can be a direct activator or repressor depending on the gene, essentially via binding to remote gene enhancers. Fra-1-recruiting enhancers most probably collaborate with non-Fra-1-recrutiting enhancers within chromatin hubs that may, at least in certain cases, include more than one Fra-1-regulated gene promoter. Finally, at least at the Fra-1 regulated gene loci we studied, Fra-1 is unlikely to be a major actor of overall chromatin 3D architecture control. As diverse AP-1 components play major roles in many cancer types, a major question will be to clarify whether the above conclusions also apply to them.

## DATA AVAILABILITY

Transcriptome, ChIP-seq and NG Capture-C data are available on the GEO database, accession numbers GSE146825 (https://www.ncbi.nlm.nih.gov/geo/query/acc.cgi?acc=GSE146825).

The three linked subseries on transcriptome, ChIP-seq and NG-capture-C can be found respectively at: https://www.ncbi.nlm.nih.gov/geo/query/acc.cgi?acc=GSE146823.

https://www.ncbi.nlm.nih.gov/geo/query/acc.cgi?acc=GSE146822.

https://www.ncbi.nlm.nih.gov/geo/query/acc.cgi?acc=GSE146824.

Fra-1 and Fra-2 ChIP-seq data are available on GEO database, accession number GSE132098 (https://www.ncbi.nlm.nih.gov/geo/query/acc.cgi?acc=GSE132098).

p300/CBP ChIP-seq data in MDA-MB-231 cells transfected with either the control siRNA or the siRNA directed against Fra-1 are available on GEO database, accession number GSE163304.

https://www.ncbi.nlm.nih.gov/geo/query/acc.cgi?acc=GSE163304.

## Supplementary Material

gkab053_Supplemental_Files
