# Supplementary material for: Fra-1 regulates its target genes via binding to remote enhancers without exerting major control on chromatin architecture in triple negative breast cancers
Source: Nucleic Acids Res. 2021 Feb 3;49(5):2488–508. doi: 10.1093/nar/gkab053 (PMC7968996; doi:10.1093/nar/gkab053)
Supplement: gkab053_Supplemental_Files [file gkab053_supplemental_files.zip › Supp Data 201221.pdf]

Supplementary Data S1: Supplemental methods for transcriptome, RT-qPCR, ChIP-seq, NG Capture-C analyses, ChIP and ReChIP

(A) Transcriptome analyses. To eliminate the bias linked to siRNA transfection itself, as well as potential bias linked to unexpected effects of control siRNA (siCTL), we introduced in our experiment another control, i.e. non-transfected cells that have received the transfection reagent (NT). The analysis of our Affymetrix data were conducted in 3 steps.

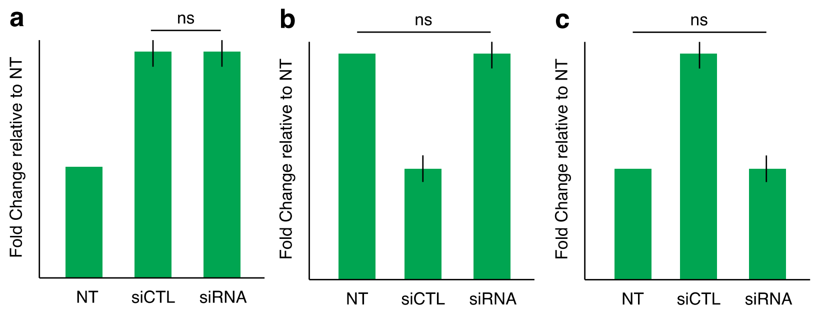

Figure A: Schematic representation of transcriptomic results indicative of false positive genes either due to siRNA transfection (a) or to siCTL bias (b and c).

In a first step, we identified the genes that were differentially expressed with both a Fold Change (FC) >1 or <-1 and a False Discovery Rate (FDR) <0.05 between (i) specific siRNAs (siFra-1, siFra2 or siFra-1+siFra-2)- and control siRNA (siCTL, Ambion #4611) transfection conditions, (ii) specific siRNA (siFra-1, siFra2 or siFra-1+siFra-2) transfection and non-transfected cells (NT) and (iii) siCTL-transfected and non-transfected cells. As mentioned in Materials and Methods, siCTL and NT samples were different for single and double transfection experiments due to the amount of siRNA transfected in each condition (4.5 nM for single- and 9 nM for double transfections). Then, we crossed the different lists of genes as follows: for single tranfections siFra-1 versus NT, siFra-1 versus siCTL, siFra-2 versus NT, siFra-2 versus siCTL, and siCTL versus NT, and for double transfections, siFra-1+siFra-2 versus NT, siFra-1+siFra-2 versus siCTL and siCTL versus NT. This allowed for the removal of, not only genes whose mRNA variations were due to a mere transfection effect (see Panel a of Figure A for illustration), but also those whose mRNA variations were due to siCTL off-target effects (See panels b and c in Figure A for illustration). Importantly, the latter category of genes is non-pertinently considered as differentially expressed when transcriptomic study conclusions are just based on the comparison between specific siRNA and siCTL conditions. In addition to

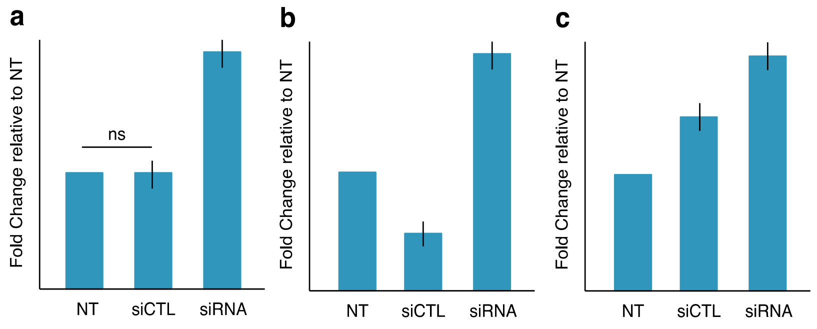

Figure B: Schematic representation of transcriptomic results obtained for gene up-regulated specifically by siFra1, siFra-2 or siFra-1+siFra-2.

genes the expression of which was clearly dependent on siFra-1, siFra2 or siFra-1+siFra2 and not affected by siCTL- (see Panel a in Figure B), we also kept two other groups of genes at that stage. These were: (i) the genes for which the siCTL effect was opposite to those of siFra-1, siFra2 or siFra-1+siFra2, as the bias induced by siCTL did not affect the qualitative interpretation (induced or repressed) of the data (Figure B, Panel b for illustration) and (ii) the genes for which the absolute FC value for siFra-1, siFra2 or siFra-1+siFra2- relative to NT conditions was higher than that of siCTL- relative to NT (Figure B, Panel c for illustration), as, in this case, siFra-1, siFra2 or siFra-1+siFra2 had stronger effects than siCTL on mRNA steady-state levels. Following this approach, we eliminated 66% (2037/3098) of the genes identified for siFra-1, 82% (1027/1253) of those identified for siFra-2 and 60% (4277/7024) of those identified for siFra-1+siFra-2, as they corresponded to genes not specifically regulated upon Fra-1 and/or Fra-2 down-regulation. In conclusion, our analysis indicated that the majority of genes (64.5%) identified as expression-varying when compared to siCTL condition only were false positives. Therefore, in addition to the required siCTL condition, the use of an additional control in the experimental setting, i.e. cells that have received the transfection reagent without any siRNA, would prevent the selection of false positive genes in transcriptomic analyses. Proceeding so allowed us to end up with 3 lists of genes significantly regulated by siFra-1, siFra-2 or siFra-1+siFra-2.

In the second step of our analysis, these 3 lists were crossed. The genes whose expression was modulated by siFra-1 or siFra-2 under simple depletion conditions but not upon transfection of siFra-1+siFra-2 (*E* and *F* subgroups in Figure 3), were eliminated. They represented 11.5 % of siFra-1- and/or siFra-2-regulated genes. The crossing of lists identified 5 categories of genes (Figure C): (i) those preferentially regulated by Fra-1 (group A) or (ii) by Fra-2 (group B), (iii) those redundantly (group C) or (iv) complementarily regulated by Fra-1 and Fra-2 (group D) and (v) those regulated antagonistically by Fra-1 and Fra-2 (group G) (also legend to Figure 1B for detailed definition of the different groups).

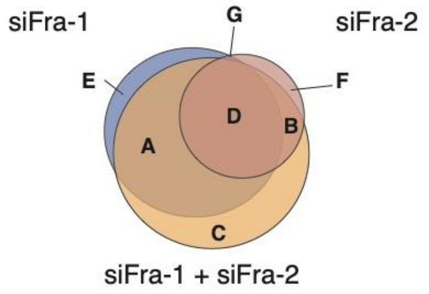

Figure C : Classification of Fra-1 and/or Fra-2 regulated genes in 5 categories upon crossing of the transcriptomic lists : preferentially regulated by Fra-1 (A), by Fra-2 (B), redundantly (C), complementary (D) or antagonistically (G) regulated by Fra-1 and Fra-2. Genes regulated by Fra-1 or Fra-2 but not recovered upon both Fra-1 and Fra-2 depletion (E and F) were discarded.

In a third step, we applied FC thresholding to these different gene categories. For the genes regulated only upon Fra-1+ Fra-2 depletion (group C, i.e. present in just one list), we retained those showing FCs with an absolute value ≥ 1.5/-1.5 or ≥ 2/-2 relative to NT. For the genes present in 2 lists (groups A, B, D, G), we retained the genes having absolute values of FCs of ≥ 1.5/-1.5 or ≥ 2/-2 in only one of the 2 lists. This avoided to lose genes with absolute FCs lower, but still very close to 1.5 or 2, in one of the 2 lists. The few

genes found to be antagonistically regulated by Fra-1 and Fra-2 (subgroup G) were not present after FC thresholding.

(B) Sequences of primers used for the amplification of cDNA by qPCR.

Gene	Primer sequence	Sense
MMP1	CATCGGCCCACAAACCCCAAA	Forward
	CCGGGTAGAAGGGATTTGTGC	Reverse
CD68	ACTCACAGGACAACCACCACA	Forward
	GTGAGTGGCAGTTGAGGGTCC	Reverse
SSFA2	AGGGTTGTGTCCTCTGTCAAT	Forward
	ACCCGCTTTTCCAATTCCTGC	Reverse
PRKCDBP	CAGGACACCGAGGAAGATCCC	Forward
	TTTGGGACAAGGCACAAGCAC	Reverse
TMEM158	TCCCTGTGTCCGTCCTGTGTC	Forward
	TCAAAGGCGCTGGGGTCTCTC	Reverse
CDCP1	GCAGTTTTCCATCCCTCGCCT	Forward
	GCAGAAGGTTCCGATCCTGAC	Reverse
HMGA1	CCCAAGCCCCATCTCATCCTG	Forward
	GCTGTCCAACCTAGTACGGGG	Reverse
ARNTL2	GGTTACTTGAGAAGCTGGCCT	Forward
	TCCATTCACTGCAAACCGGGT	Reverse
HGS	CCAGACCCACCTTTTGTTCCC	Forward
	ACCCAAGCTCCTACCCCCAAA	Reverse
PTPRE	CAGCTCAAGGTCCCAAACAGG	Forward
	TTCCATAGGTCCAGCAGCCTT	Reverse
CLU	TGGAGAACGACCGGCAGCAGA	Forward
	GGACGATGCGGGACTTGGGAA	Reverse
KRT80	CTCCAAGGTGACTGTGAACCC	Forward
	GCCTTCATCTCCTCCTTCTCC	Reverse
BSFP1	AGCAAACCCTGAAGAATGAGC	Forward
	AACAGGGGAATGGGAGTTTCA	Reverse
KRT7	CAGGAGGAGAGCGAGCAGATC	Forward
	ACATCCTTCTTCAGCACCACA	Reverse
HES1	ACTGATCACCAAGTAGCCACA	Forward
	CACCGGGGACGAGGAATTTTT	Reverse
TGFB2	TTGCAGAACCCAAAAGCCAGA	Forward
	TGTCGATGTAGCGCTGGGTTG	Reverse
ID1	TGGACGAGCAGCAGGTAAACG	Forward
	CGGGGGTTCCAACTTCGGATT	Reverse
SMAD6	GGTATCAACAAGCCTGCCTTT	Forward
	AAATGTGTCTGGAAAGCCCTG	Reverse
TES	TCCCAGAGAGGTGAAGGAGAT	Forward
	TGCTGCTGGGGTGCTTCTATC	Reverse
PLCE1	GACATGATCACCAGGGAGGAA	Forward
	CTACTTTCATCTGCAGCCGAC	Reverse
S26	CTGCACTAACTGTGCCCGATGCGTG	Forward
	GACGCTCGCTTCAGAAATGTCCCTG	Reverse

(C) ChIP-seq thresholding parameters for peak calling. Thresholds were set to values allowing non-ambiguous signal-to-noise ratio, thus avoiding false positive peak detection. The regions separated by a distance lower that the defined Max gap values were merged into a single region. Classically, the chosen values depend on the type of the ChIP signal. Min Run defines the minimal width of the region that must meet the threshold to be annotated as a peak.

	Threshold (by Value)	Max Gap	Min Run
Fra-1	50	50	50
Fra-2	50	50	50
H3K4me1	30	1000	250
H3K4me3	30	500	150
H3K27ac	50	500	150
p300/CBP	60	50	50
Pol II	50	800	100
CTCF	50	50	50
p300/CBP (siCTL or siFra-1)	25	50	50

(D) NG Capture C capture probes used for the 35 studied genes. One or two 70-mer biotinylated DNA oligonucleotides were designed at right and/or left ends of restriction fragments containing the promoter of interest using the CapSequm online tool (http://apps.molbiol.ox.ac.uk/CaptureC/cgi-bin/CapSequm.cgi).

Viewpoint	Oligo	Probe Sequence (5'-3')
FOSL1	Left	GATCCCAGGAATTCAGCCCGAAGCGGAGAGACCCTCCTAGCCTTCGA GGCGACCACCCTAGGCGCCCACG
	Right	GGAGTTCGCGGGTCTCTGCTGCCCGCTGCGCGGTGGGGGCAACTGG CCCCGGACGGCGAGGGGCAGGATC
MAP2K1	Right	GGCGGTGAACCTCGGGGCCCGGCTGGGGAGGCCCGAGCCGGGGAG CAGGAGCGCGCGCCAGGCTCCGATC
ZNF114	Right	AGAGCCTAGGAACGGTGGCGCGGGCGGAGGCGACAGCGAGGACCCA AGGCTGGGGTTTCCTGCTTGGATC
CDCP1	Left	GATCGGGGACACTCTCACGTGCCGAGGGCAAGGTTGACTCCAGGAGG GCAAGGCCCACGCGCCCGACGCG
	Right	GCGCTCCGCCCTGGCTCACTCACCTGCGCGCGGGCGGACCGGCCCG AGCCCCGCCCTGCGCAGCAGGATC
SSFA2	Left	GATCTTGGGGATATTAGTAAACCATTAGAAGATGTCTTGGAATTGTAAA TCTAGAGCAATGCGCTAGAAA
	Right	GTGGCGAGTCGCAGGAGGAAGGCCTGGGCCAAGTGCCGCAGCTCCT GGCAAGCGTCGGAGACGGAGGATC

PDE2A	Left	GATCGGGGAGTGGGTCCCACAGAGAAAGCCAGTGAAGCCCTGATGGA AGGAGAGATTTGGACTCTGGGGT
	Right	ACAGCTCTCAGTGTGCACTGCGGTCCCACCGTGGACTGGAAGCTCAG TGCTTGGGGTGGAGCCCCGGATC
PITPNC1	Left	GATCTCTCCTCTGAAACCAGGCAAGGGTTTGTTCCCAAATCCCACCCA TTGCCTCTCCAGCCCCAGACTT
	Right	GGAGCGGCGCGGGGACCGGCCCTAAAAAGAGCAGGAAATCTTGTTTA CCGCCCGCGGAGAGAAGCCGATC
MXD1	Left	GATCCTCCTGTTTTCTAGAAGCTGAACATGGTTATGCCTCCATGTTACC ATACAATAACAAGGACAGAGA
	Right	TCTTTTACTTTTCATAGTAATGATTTTATAAACACATTAAAGTACCTGAC TTGGCATAAGTGATAGGATC
RPSAP52	Left	GATCTAGAGGCCCCTGAGTTTTCCCGCTCTGCCCAAGCGACACTTTAC ACCGCATTTGAATCCTGTTTTA
	Right	TTAGTCTCACTTAATCACCGAATCTGAATGAAATGTGAATAGATGATTC ACTGGATAAATATTTGGGATC
HK2	Left	GATCTCAGGAGGTGTCTAAGGGCATGGAGATAAGTGGTCAGATGCAC GGTCTGTTTTATAGGTGGAATTA
	Right	TGGGGTTGGAGCTTCCACTCCTCTCAGCATTGGTAAGCCTCCTCACCC ACCCCATCCCATGTCCAAGATC
EMP1	Left	GATCCTCTAAGAACCAAGTCCTTGAAACTTTTGGCTCAAAGTGGATACA GAGACAACTTTTTCTAGAAAG
	Right	TGGCAGTGACCGGGTTCTTGTTCTAGGATGTGGAGGGAGCAGGGACT TTGAAACAAACTGGGATAGGATC
HMGA1	Left	GATCATGGCTCGCTGGTGGCCATTAATAAAACACTTTGGATTTCACAA GTTTCACGTTTGAATTTCACAA
	Right	GGGGGCCTGGGCGGCGAGCACGCGGCGGCGGCGGTCTCTGAGCGC CTCTGCTCTCTCCCGGTTTCAGATC
MYO10	Left	GATCCCCGCGCGAGCGCTTGGAGGTGAGCAGTCCCGCGCCGCGGCT CAGCCAGCTCTCGCCCGCGGGACT
	Right	GAGGAGAAGGGACTGGGGGCTTCTGTGCCTTTCATGTTTAGCAGACC CGGGAACCGGCTCAACACGGATC
SOX9	Right	TCGGGCTCCGGCTCGGACACCGAGAACACGCGGCCCCAGGAGAACA CGTTCCCCAAGGGCGAGCCCGATC
SRGAP1	Left	GATCCCCTGGGATTACCGACGGAGCTCAATTTCGAGAGTATGGTGCCA GCGCCCGGGCTAATCTAGTCCT
	Right	CCATTGAAAGCAAACCCGGAACAGCTGGATAATGTCCACCCCGAGCC GATTCAAGAAGGACAAAGAGATC
ASB1	Left	GATCCCAAGGGCAACACGTGGCAGGGAAGGTGGAGGTGCAGGTGCA GGAGGGCCACTCTGCTCAGGGACT
	Right	ATACAGATTTGATATGACCCAACCGCAAAAGTAGGACGTGGGCCACCT TCCAGCTCTGAGCGCACGGATC
CD44	Left	GATCTTGCTCCAGCCGGATTCAGAGAAATTTAGCGGGAAAGGAGAGG CCAAAGGCTGAACCCAATGGTGC
	Right	AGCCTCTGCCAGGTTCGGTCCGCCATCCTCGTCCCGTCCTCCGCCGG CCCCTGCCCCGCGCCCAGGGATC
LIPH	Left	GATCGTGTGTCACATTCACAAGAATGATTTACTTCGCAGAGGCTTAAGA GTTTCCACTGTGGGATTTTGC
	Right	CTCAGAGGTGTGGTGACAACAGGAAGTTCCCTAGGGACTGACTGCAG GCAGTCCCAACCCTTTCAGGATC
TGFB2	Left	GATCGTGATGTTATCTGCTGGCAGCAGAAGGTTCGCTCCGAGCGGAG CTCCAGAAGCTCCTGACAAGAGA
	Right	GGTCTAGGGAGTCATCCAGGAACAAACTGAGGGGCTGCCCGGCTGCA GACAGGAGGAGACAGAGAGGATC
IL8	Left	GATCAAAGAAAACTTTCGTCATACTCCGTATTTGATAAGGAACAAATAG GAAGTGTGATGACTCAGGTTT
	Right	TTCTGCAGCTCTGTGTGAAGGTAAGCACATCTTTCTGACCTACAGCGT TTTCCTATGTCTAAATGTGATC
ID1	Left	GATCAGGGTCCGAGAAGCATCTTCCAAGAGGGTCCGGAAACCCAGGC TCTCTGGGCTATCCAGAGCCAGC
	Right	CGAAGTTGGAACCCCCGGGGGCCGAGGGCTGCCGGTCCGGGCTCCG CTCAGCACCCTCAACGGCGAGATC
TSPAN2	Left	GATCCCCAGTCCCCAGGCCCGCGCTACGAGCGCGGGGAGCGGCAGG CTCCGGCGGGGAGGGGGCGGAGCG
	Right	GGCGGGGAGGGGCGCGTGGACCCCAAGCGGCGTGGCTGGCGACGG GTGGGGGCGCCAGCGGGTGGAGATC
HES1	Right	TGGGAAGTTTCACACGAGCCGTTCGCGTGCAGTCCCAGATATATATAG AGGCCGCCAGGGCCTAGGGATC

KRT80	Left	GATCGATGCTCAGGGAAACACAGAGCTCAGCTACCGGCCCAGGCCTG GCAGCCCTTCATGTTTGGGACCC
	Right	CAGCTCTCCCAGCCCTGCGTCTCCTTTCCTGGCCTGGTGGTCCTGATG TTCTGGCCCTGGTGGCTGGATC
HEG1	Left	GATCATAAGAAAGAACTGGACACAAAGGTAGCAGGGAGAAGAGGTCA GTTCCTTCTCTCTGCGTGGGACA
	Right	GCAGGGAAAAGAAAACTGTGAATTCAGGCAGGCTAGGGCAAACTCAT CATCAGGAAAAGAACTGCAGATC
SMAD6	Left	GATCCCTCACCTACAGCCCTCTCCGAGGTGCGGAGAGTAAACTTGAG AAGGGAGAGGAAGCACCCGGCCT
	Right	TTTGGCCCGGCAGAGCGACCCCCGACTTTGTGCAAGGAGCGTAGAGC CCCTAGTGCAGTCACGGAAGATC
EDN1	Left	GATCTGCCAAAATAGTCTGACCCCCAGTAGTGGGGCAGTGACGAGGG AGAGCATTCCCTTGTTTGACTGA
	Right	GCTCGCTGCCTTCTCTCCTGGCAGGCGCTGCCTTTTCTCCCCGTTAAA AGGGCACTTGGGCTGAAGGATC
TES	Left	GATCCCAGCTCTTTAGAAGCCGGCCCGTGGACGCCCAGAGAATCCCT TCGGAGACCAGGTCAGGGTCACT
	Right	CCGCGGGAGTTCCGCAGGTTTCCCGTGTTCGCAGCGGAGCCGGAGG CCAGCTGAACCCGGCCGTGGGATC
TMC6	Left	GATCGCGGCCGCTCACCTGGGAGGCGCCGGGGAGGAGGGGCCGCG CGCGCAGCCAGGGCTCACCTGTGCC
	Right	CGCTCAGTTTCCCCAAGTTCCAAAAAGGGGCAATATTTCCAGACTCAT CTGCTTCCCATGGTTAGGGATC
ABTB2	Left	GATCCGTGGCCAGCAGGAGCCCCACGCTCCCGGCGTCCCGACTCGC AGCTGCCACTGGAAAGAACCCCGT
	Right	TCCGACATGCAAATAACCCCGGGCGGCTTACCAATCACCGGCCGCGA TGGGCGGGGCTGCACCAATGATC
GALNT2	Left	GATCGTTTTCTTGTATCGTTAAAGATGCTTAATGTGCTTTTTAAAGAACG CAACGAATGAGAAAGAAAAG
	Right	CACTCCCCGCTTCAGGGAGAATCCCTCTTCTGTGGCTGTGTTTTTAAC ATGCTCCAGGGGCCGAGTGATC
FNDC3B	Left	GATCTATTGGATTGTACCCTATTGGCACTAAGGAACTACTGCCTACCC CCCGCCCCGGCTTCTCATGCAG
	Right	AATGAAAAAAAGTCAGTTTTAGGCTTAATTATCACTGAGTAAACATTGC CTTTATATTACTTTGGCGATC
TNS3	Left	GATCTCTGTGCTCCCCAAGCCTCGTTCCTGCGCCCGGAGCGCGAAAG CCCGCGCCGGGCCCGAAACAAAG
	Right	CCAAGTGGTCTGGAGGCTTGCAGGTTCCGCCGGGCCGGTGTTAGAGT ACCTGGGGAGGCGGGTTCGGATC
LRP10	Left	GATCTCAGACCCTGGGACAAGAAAAGTACTGAGAGCTTAGGGCCCAA AGGAAAGACATCACAGTTATGGA
	Right	GGCCCCAACCCCCAGGAAGTCTTCAGCTGCTCCGCTGGGCCCTGGTC CTCTCTCCGGCTTCCCCTAGATC
TCF7L2	Left	GATCATTGTTAGCCGCCCCCGCCCCGCCCACCCCGGCTGTTTATTTAT GCACACGTCACTGGGCCGGCCC
	Right	GGCAGTGTGTTCCTCTCGCCCTGTCAATAATCTCCGCTCCCAGACTAC TCCGTTCCTCCGGATTTCGATC

(E) Sequences of primers used to amplify by qPCR Fra-1 binding sites at the HES1, RPSAP52 and SSFA2 PIRs.

Amplicon	Amplicon Position to TSS (kb)	Primer sequence	Sense
HES1	-46.3	TTGCTGCGGGGGAAGCCA	Forward
		GTCAGTGGAGCTCGGTGG	Reverse
HES1	-130.9	GAGCCGAGACAGTGCCAC	Forward
		GAGTGGCCTTCTGTCCGG	Reverse
HES1	-132.2	GCAGCTGAGTCATCGAGG	Forward
		GGATCATGCGGCCTGCTG	Reverse
HES1	-263.9	AGGTTTCTATCAGCCTCCCAG	Forward
		CCAGGGCGCTAAACCACAA	Reverse
HES1	-266.2	CGGCCACAACAGGACCTT	Forward
		ATTCCTCCCCTCACCCCG	Reverse
RPSAP52	+47.7	GCCCAGCCTTCTCCCTGA	Forward
		CCCACCAAAGAGCCACTG	Reverse
RPSAP52	+163.1	TTTCTCCGTCAGTGCCCC	Forward
		AGCTGCGGGTGGGTGTTT	Reverse
RPSAP52	+177.8	CCCTGGCCGTTTGTAGTA	Forward
		TCACTGCTTCGTCTGGCT	Reverse
RPSAP52	-191.8	AGAGTTTTGAGACGCTTAGCTG	Forward
		CTACGGTCCATTCCTCTGCTT	Reverse
RPSAP52	-195.1	CACTAAGGCAATCGGCACT	Forward
		CTTCCCAACCCTCAAACCGAA	Reverse
SSFA2	-80.83	CCAGGCCCTTTAACATAGGCTC	Forward
		GTCTTAACTCCCCGTGCTGT	Reverse
SSFA2	-100.44	AGGGGGAGACACTGCTTT	Forward
		ATGGCCCCGTGAGTCATT	Reverse
Negative control	NC	TCAGGGGCCGCAGGAACG	Forward
		CCGCATCAACCCCCCTTC	Reverse

(F) **Fra-1- and Fra-2 ChIP-ReChIP analyses.** For the first ChIPs, $50x10^6$ cell samples were fixed using a 1% solution of paraformaldehyde (Euromedex) at room temperature (24°C) for 7 min. Fixation was stopped by adding glycine to a final concentration of 125 mM. After two washes with PBS, cells were scrapped and incubated on ice for 10 min in cell lysis buffer (PIPES pH7 5 mM, KCl 85 mM, NP40 0.5%, NaButyrate 10 mM + protease inhibitors). Nuclei were recovered by centrifugation (5,000 rpm for 10 min at 4°C). They were then lysed in 1.2 ml of Nuclei Lysis Buffer (Tris-HCl pH7.5 50 mM, SDS 0.25%, EDTA 10 mM, Na Butyrate 10 mM + protease inhibitors) at 4°C for 2 hrs. Next, lysed nuclei were sonicated at 4°C (10 to 15 cycles) until clarification of the lysates using the Bioruptor Pico device from Diagenode.

After sonication, absorbance at 280 nm (A_{280}) of 1/100-diluted samples was measured and A_{280nm}-adjusted to 0.15 with the nuclei lysis buffer if necessary. 0.5 ml of sonicated chromatin (corresponding to $20x10^6$ cells) was incubated after a 2.5-fold dilution in immunoprecipitation (IP) dilution buffer (Tris HCl pH7.5 50mM, NaCl 167 mM, Triton X100 1.1%, SDS 0.01%, EDTA 1.2 mM, Na Butyrate 1 mM, + protease inhibitors) with 15 µg of anti-Fra-1 antibodies (a mix of the two anti-Fra-1 monoclonal antibodies sc-376148X and sc-28310X from Santa Cruz Biotechnology, 7.5 µg each, as ChIP efficiency was better than that when using 15 µg of each antibody individually) coupled to Protein G-coupled Dynabeads. In parallel, 0.5 ml of sonicated chromatin (also corresponding to $20x10^6$ cells) was incubated with 50 µl of the anti-Fra-2 monoclonal antibody D2F1E from Cell Signaling (ref. # 19967). 3 µg of a control mouse IgG (sc-2025 from Santa Cruz Biotechnology) was added to 100 µl of chromatin (corresponding to $4x10^6$ cells) as a specificity control. After 20 hours of incubation at 4°C under rotational agitation, beads were washed twice with the ReChIP wash buffer (NaCl 500mM, EDTA 2 mM, SDS 0.1% and NP40 1%) and TE buffer (Tris-HCL pH 7.5, 10mM, EDTA 1mM). Elutions of Fra-1- and Fra-2 ChIPped chromatin samples were carried out in 55 µl of Re-ChIP elution buffer (Tris-HCl pH7.5 10 mM, EDTA 0.1mM, SDS 2%, DTT 15 mM) for 45 min at 37°C under agitation according to Truax and Greer (Methods in Molecular Biology, 2012, Chapter12, 175-188), whereas 20 µl was used for the IgG ChIP control. 10 µl of eluted Fra-1 and Fra-2 ChIP samples (corresponding to $4x10^6$ cells) were used as controls of the efficiency of first ChIP step of our ReChIP experiments through comparison with the control ChIP conducted with the control mouse IgG. For the ReChIP step, 40 µl of Fra1- and Fra-2-ChIPped samples (corresponding to $16x10^6$ cells) were diluted 20-fold in IP dilution buffer (800 µl final) and 200µl of diluted chromatin (corresponding to 4.10^6 cells) were incubated in the presence of either 3 µg of anti-Fra-1 antibody, 10 µl of anti-Fra-2 antibody, 3µg of control IgG or empty beads, the latter two samples taken as negative controls. After overnight incubation at 4°C under rotational agitation, beads were classically washed with low salt-, high salt-, LiCl- and finally TE buffer. Then, ReChIPped samples were eluted in 250 µl of a buffer containing $NaHCO_3$ 100mM and SDS1% under agitation for 45min at room temperature. After decrosslinking-, RNAse 1- and proteinase K treatments, DNA was purified using the gel and PCR clean-up kit from Macherey-Nagel. Purified DNA samples were then analyzed by qPCR using the LC480 device from Roche (45 cycles at 94°C, 70°C and 72°C) using the primers described in Supplementary Data 1G. 10 ng of MDA-MB-231 cell line genomic DNA were used as a standard for relative quantification using the LC480 Roche software. The data were then normalized to the chromatin input taken prior to ChIP.

(G) Coordinates of the F1F2 peaks and sequences of the primers used for qPCR amplification after Fra-1 and Fra-2 ChIP and reChIP experiments

Amplicon	Coordinates	Primer sequence	Sense
NC1	chr6:43,971,412-43,971,804	CCATGTCAGGTAGGGTGAGAG	Forward
		TTTGGGGGAAGGAGCTGATGG	Reverse
NC2	chr1:218,517,559-218,517,792	AGGCCCCATACACAACTGAAG	Forward
		AGCCATACTGACCAGACAGAT	Reverse
NC3	chr6:34,207,747-34,207,973	CAGTTGGGCAGAGGAGGGGTA	Forward
		AGGGAGGAGATGGTTGAAGCT	Reverse
A	chr6:34,211,925-34,212,187	CATTCCCGCCCAGTGAGTGAG	Forward
		TGCAGGGGAACCAGGTCCTTG	Reverse
B	chr8:67,036,231-67,036,385	TTCCAAGACCACAAAGGGAGG	Forward
		AAGGAGGAAATGATCCCTGGG	Reverse
C	chr8:128,866,521-128,866,713	AGCAGTGAGGCCTCTGAGTCA	Forward
		CCCCTTTAAATGCTCTGAGGG	Reverse
D	chr4:26,199,054-26,199,236	GGAGAAAGTGGGCCAAGGAGC	Forward
		CTCTCTTGCCGCTTTAGGGGA	Reverse
E	chr8:94,892,629-94,892,784	GCTGAGCAATTGGGGAAATGA	Forward
		CCCTTCCGCATGCCAAACTTT	Reverse
F	chr2 :9,320,027-9,320,306	CTCCGCGTGGTGGAAAAAGCA	Forward
		CCCAGTGACTAATCCTCCCAC	Reverse
I	chr2:9,374,893-9,375,152	CTCCCACACCTTCCTTCCAGT	Forward
		CGTCTACTCCTTGCACCAGTC	Reverse

(H) Positions of the F1F2 peaks in cAEs amplified after Fra-1, Fra-2 and p300/CBP ChIP in MDA-MB231 cells transfected with siCTL, siFra-1 or siFra-2, Fold changes (siFra-1/siCTL) in ChIP-seq and sequences of the primers used for qPCR amplification.

Amplicon	Coordinates	p300/CBP FC siFra1/siCTL	Primer sequence	Sense
A	chr8:128,866,351-128,866,801	1.07	AGCAGTGAGGCCTCTGAGTCA	Forward
			CCCCTTTAAATGCTCTGAGGG	Reverse
B	chr4:26,198,951-26,199,351	1.04	GGAGAAAGTGGGCCAAGGAGC	Forward
			CTCTCTTGCCGCTTTAGGGGA	Reverse
C	chr8:94,892,551-94,892,851	1.12	GCTGAGCAATTGGGGAAATGA	Forward
			CCCTTCCGCATGCCAAACTTT	Reverse
D	chr16:48,161,051-48,161,301	8.84	CTGGGAGTCTGCTGCTGAGTT	Forward
			AGGCAACGCTGTGGGTTTAGG	Reverse
E	chr3:58,457,101-58,457,401	5.21	CTCCTCTGTGGCCTGAACCTT	Forward
			CCCCAAACCCTGCTGTGAATG	Reverse
F	chr6:11,435,051-11,435,151	5.13	ATCCGGAGCCTAGACACTTGA	Forward
			GTCCTGATCATAGCAGCCACT	Reverse
G	chr15:63,061,901-63,062,101	-5.59	GTCTTCCTGTCCACATCCCCA	Forward
			CAAACCTTTAGCCAGCACCT	Reverse
H	chr19:39,564,301-39,564,651	-1.97	GAAGTGGTGAGGAATCCATGC	Forward
			CACCCCACACTTCACCTCATA	Reverse
I	chr12:114,165,351-114,165,851	-3.24	GAGGGACAAGACACAGGAAAG	Forward
			GTAATGACAGTGCCCATCCCA	Reverse

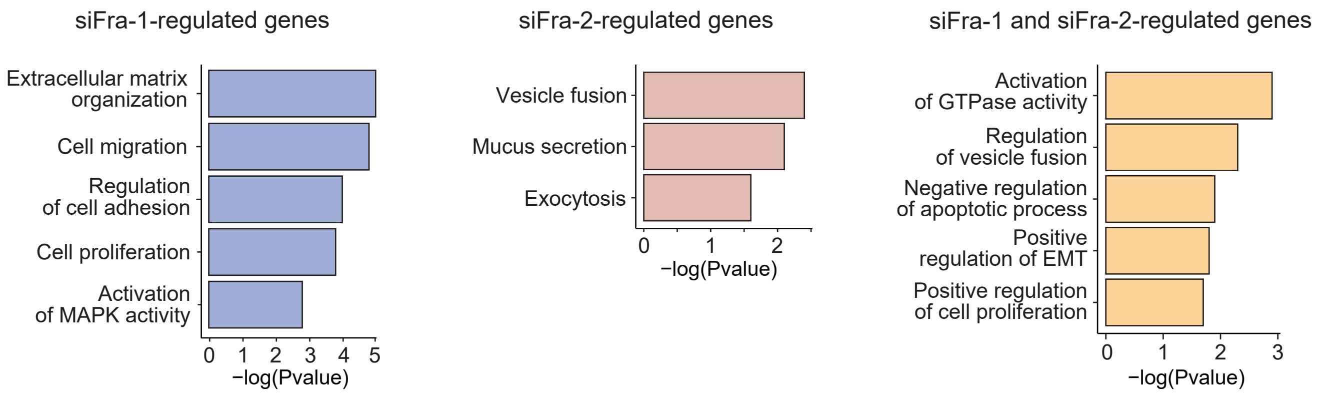

Supplementary Data S2: Biological pathways overrepresented upon ontology analysis using the publicly available DAVID software (Goterm) of genes regulated by siFra-1 and/or siFra-2 (FC≥ ±1.5). Similar pathways were found with the GeneGo software (see Figure 1D).

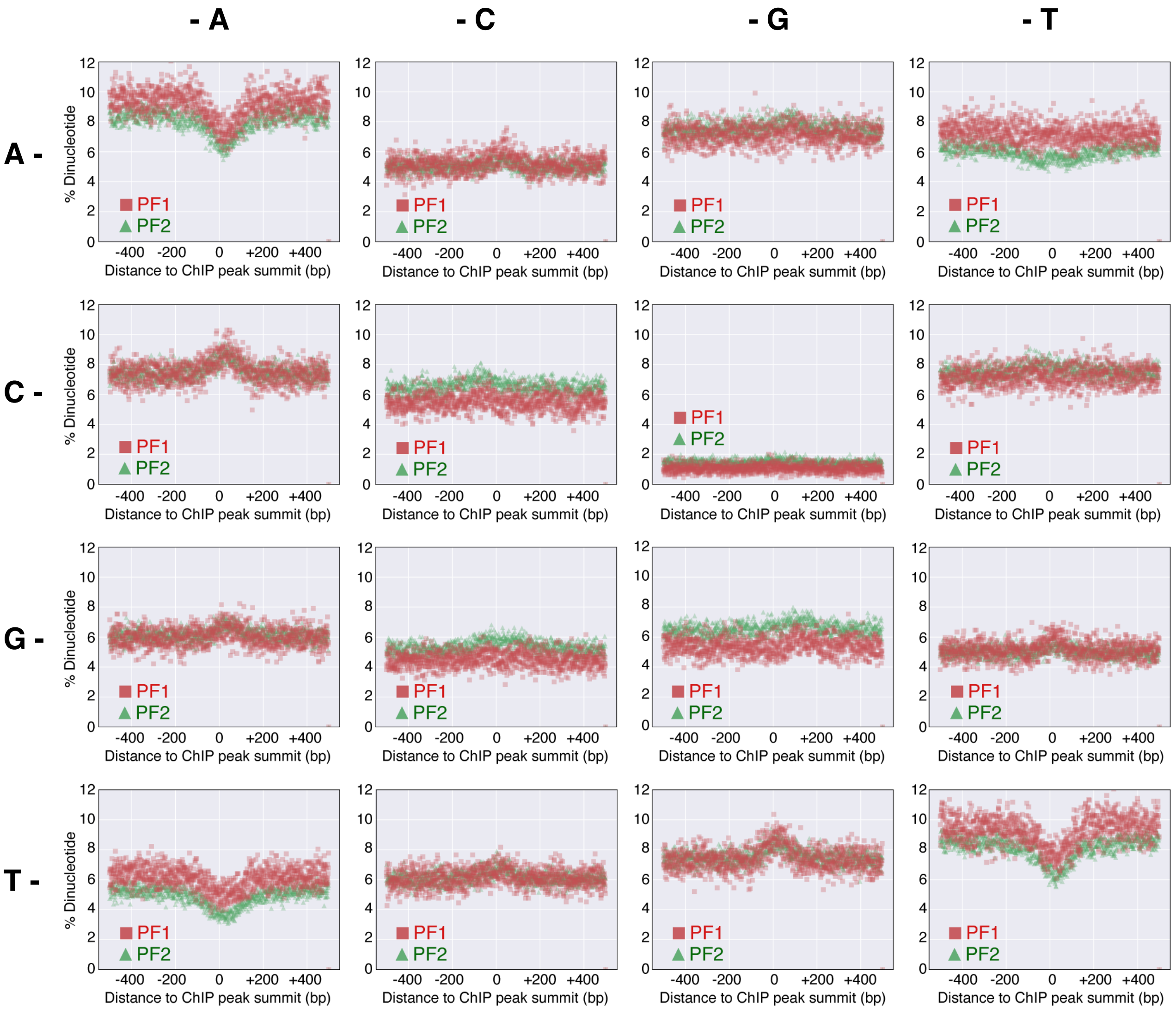

Supplementary Data S3: Dinucleotide distribution around ChIP-seq peak summits. The x-axis represents the distance to Fra-1 and Fra-2 ChIP-seq peak summits in bp. The y-axis indicates the percen-tage of the indicated dinucleotides in all sequences considered at each position (x-axis). At each position, the sum of all (16) possible dinucleotide percentages is equal to 100. As visible on the figure, PF1 peaks (red squares) present a higher frequency of AA-, AT-, TA- and TT dinucleotides than PF2 peaks (green triangles) in the ±500bp region surrounding the ChIP-seq peaks.

A **PF2 peaks**

Rank	Motif	pvalue	log pvalue	% of Targets	% of Background
1	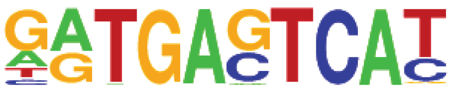	1e-1226	-2.825e+03	66.21%	16.09%
2	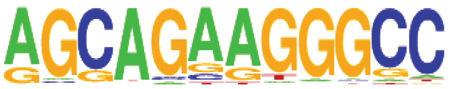	1e-93	-2.157e+02	2.39%	0.15%
3	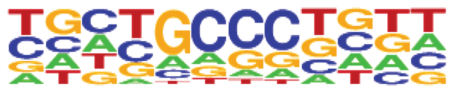	1e-87	-2.026e+02	1.85%	0.06%
4	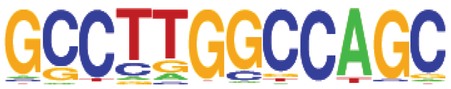	1e-74	-1.727e+02	1.65%	0.10%
5	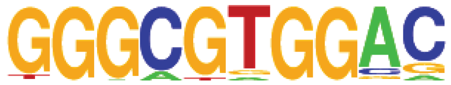	1e-73	-1.694e+02	1.63%	0.11%

B **PF1 peaks**

Rank	Motif	pvalue	log pvalue	% of Targets	% of Background
1	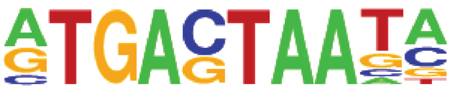	1e-88	-2.033e+02	39.55%	18.13%
2	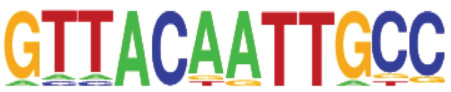	1e-26	-6.039e+01	1.63%	0.09%
3	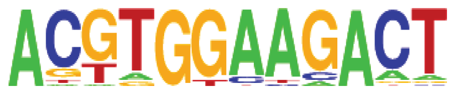	1e-24	-5.719e+01	1.57%	0.09%
4	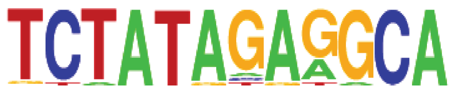	1e-23	-5.515e+01	1.13%	0.03%
5	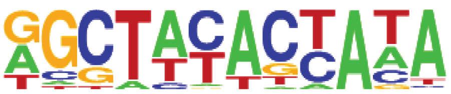	1e-23	-5.301e+01	1.32%	0.06%

Supplementary Data S4: Motif enrichment in PF2 and PF1 peaks using PF1 and PF2 peaks as background, respectively. Using the HOMER software, the canonical AP-1 motif (TGAC/GTCA) was found enriched in PF2 peaks (A) but not in PF1 peaks (B).

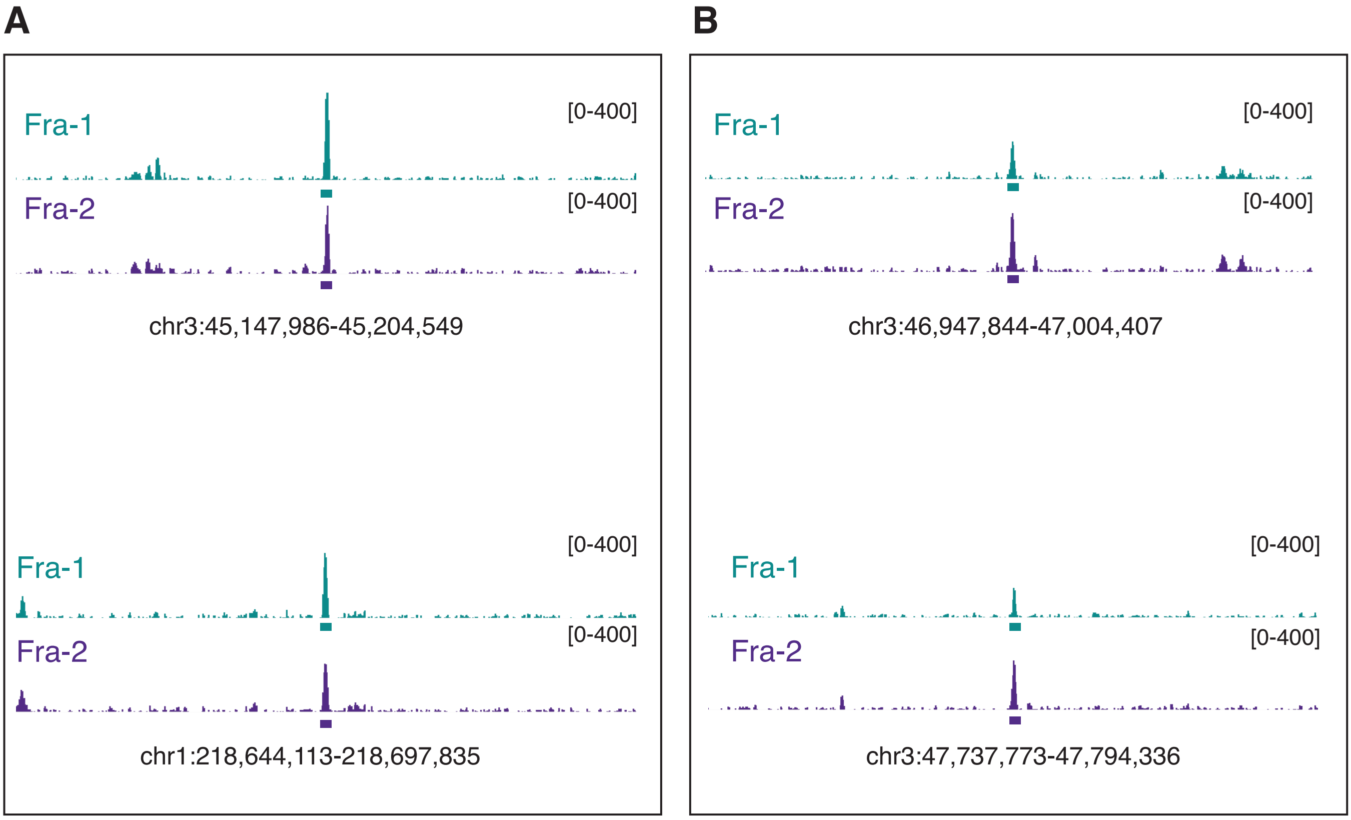

Supplementary Data S5: Examples of F1F2 peaks where (A) Fra-1 signal intensity is higher than that of Fra-2 and (B) Fra-2 signal intensity is higher than that of Fra-1.

TFcoop variable	Motif	Regression coefficient
MA0841.1 NFE2	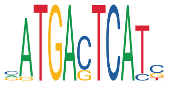	-0.26236717
AT mononucleotide	5'– A – 3'<br>3'– T – 5'	0.034804528
TpA dinucleotide	5'– T A – 3'	0.64480953
MA0491.1 JUND	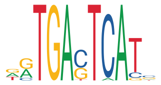	-0.21894742
MA0754.1 CUX1	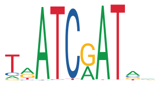	0.08557045

Supplementary Data S6: Top 5 variables selected by TFcoop to regress ChIP-seq signal at F1F2 peaks. The corresponding JASPAR PWMs and the regression coefficients calculated by TFcoop are shown in the middle and the right columns, respectively. The negative regression coefficients refer to the PF2 class, whereas the positive ones refer to the PF1 class.

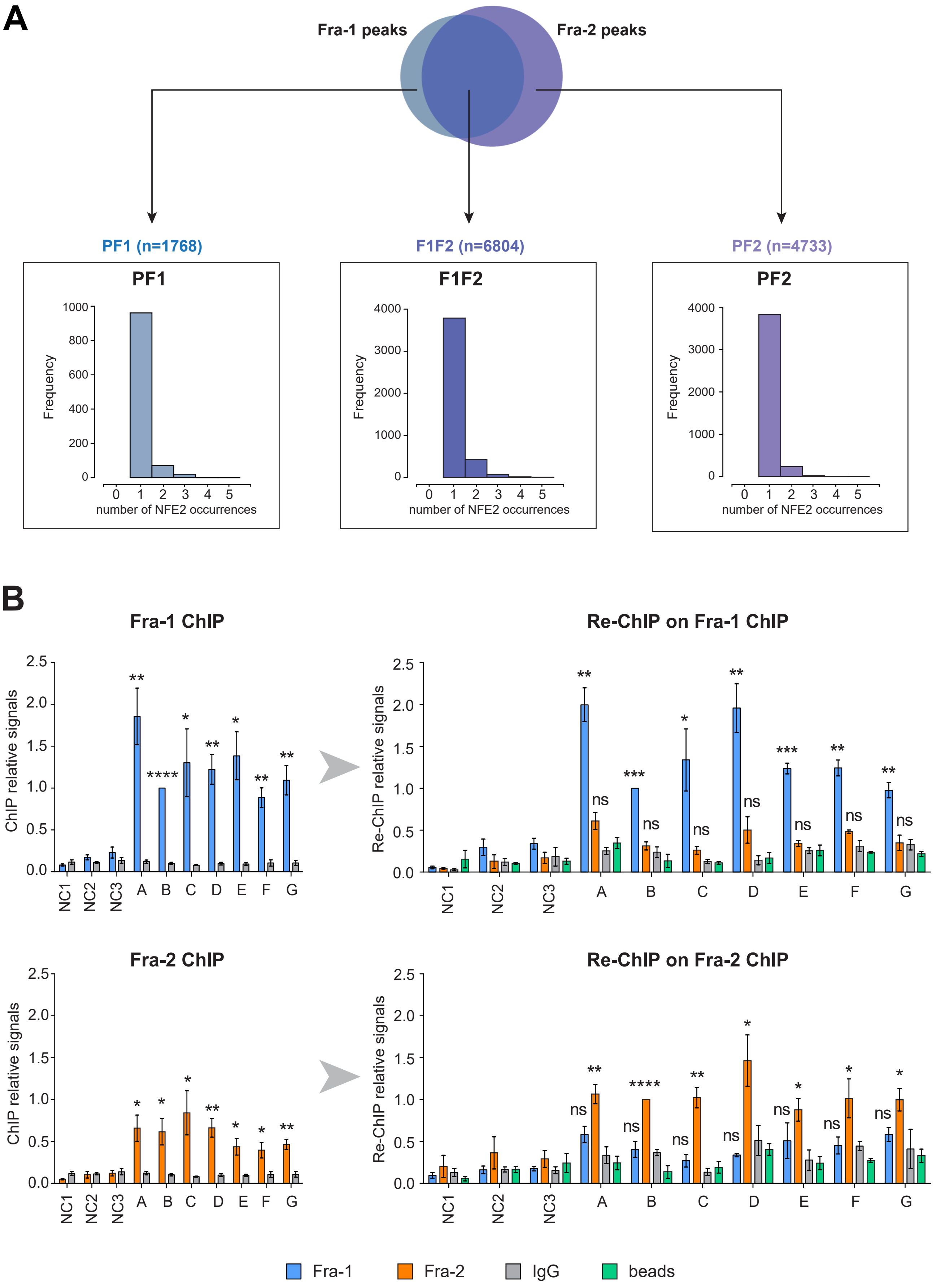
A
Fra-1 peaks
Fra-2 peaks
PF1 (n=1768)
F1F2 (n=6804)
PF2 (n=4733)
PF1
F1F2
PF2
Frequency
number of NFE2 occurrences
B
Fra-1 ChIP
Re-ChIP on Fra-1 ChIP
Fra-2 ChIP
Re-ChIP on Fra-2 ChIP
ChIP relative signals
Re-ChIP relative signals
NC1
NC2
NC3
A
B
C
D
E
F
G
ns
Fra-1
Fra-2
IgG
beads

Supplementary Data S7: (A) Number of NFE2 motif occurences in PF1 (left panel), F1F2 (middle panel) and PF2 (right panel) peaks. The number of NFE2 occurrences was found similar in PF1, PF2 and F1F2 peaks. Only motifs with a score higher than 0.8 are considered to limit false positives. **(B)** Re-ChIP experiments at F1F2 peaks. Chromatin was first immunoprecipitated with antibodies against Fra-1 (upper left panel), Fra-2 (lower left panel) as well as with control IgG. For mean calculation, signals were first normalized to input and then to peak B signal in Fra-1 ChIP, arbitrarily set to 1. After ChIP elution, Re-ChIP were carried out using antibodies against Fra-1, Fra-2, control IgG or empty beads, as described in supplemental methods (supplementary data 1F). qPCR amplification was carried out on 10 regions, 3 regions devoid of Fra-1 or Fra-2 that were used as negative controls (NC1, NC2 and NC3) and 7 regions corresponding to high F1F2 peaks located in cAEs. Signals were normalized to input and to the value of peak B signal for Fra-1 in re-ChIP on Fra-1 ChIP (upper right panel) and on peak B signal for Fra-2 in re-ChIP on Fra-2 ChIP (lower right panel) arbitrarily set to 1. Results are the mean of 3 independent experiments with technical qPCR replicates for each experiment. p-values were calculated at each position with respect to the higher values found in IgG or bead controls using a two-tailed unpaired t-test (Prism5 software). (*) (**) (***) and (****) correspond to p-values of ≤ 0.05, ≤ 0.01, ≤ 0.005 and ≤ 0.0001, respectively, ns = non significant. Coordinates of regions analyzed in B and sequences of the primers used are given in supplementary data 1G.

A

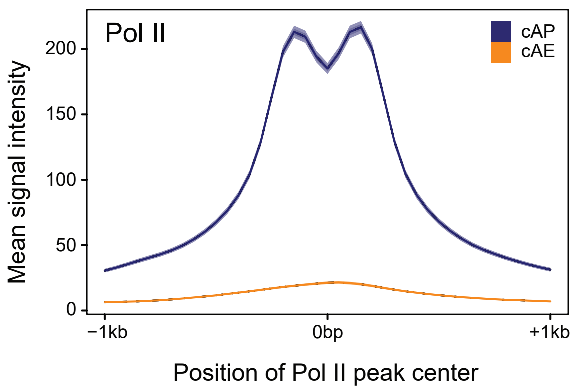

B

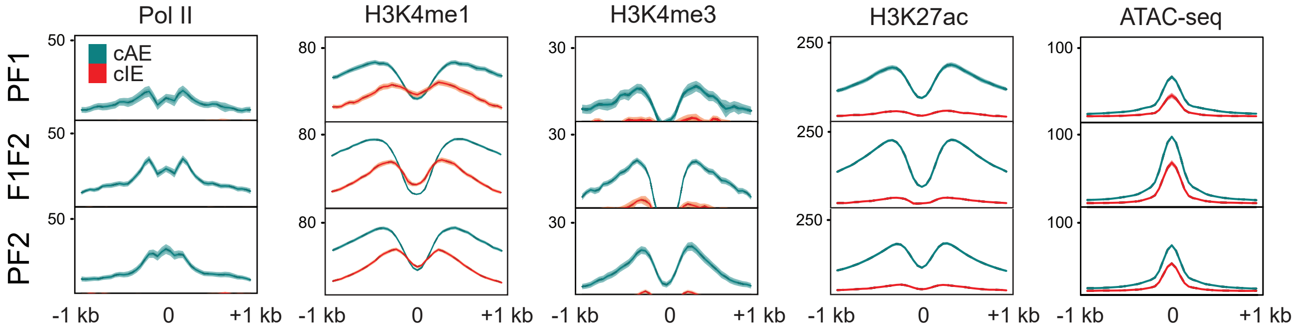

C

p300/CBP in F1F2 peaks in cAEs	number of peaks	percentage of peaks
parental MDA-MB-231 cells	2828	100%
siCTL-transfected MDA-MB-231 cells	2152	76%

D

FC = siFra-1/siCTL signal intensity	number of peaks	percentage of peaks
-1.5 < FC < 1.5	966	45%
FC ≥ 1.5	543	25%
FC ≤ -1.5	643	30%

Supplementary Data S8: **(A)** Pol II metaprofiles at cAEs and cAPs. Consistently with the available literature (see Andersson and Sandelin (50); Benton et al. (51);Gasperini et al. (52); HO et al. (53 in main Text and references therein for more information) Pol II signals are much higher at cAPs (promoters) than at cAEs (enhancers). **(B)** Comparison of ChIP-seq and ATAC-seq metaprofiles at PF1, F1F2 and PF2 peaks between cAEs (green) and cIEs (orange). **(C)** ChIP-seq peak overlaps. p300/CBP peak intersection at F1F2 peaks in cAEs in parental MDA-MB-231 cells and in siCTL-transfected MDA-MB-231 cells. **(D)** Number of p300/CBP peaks at F1F2 in cAEs according to the Fold-change in signal intensity between the siFra-1 and the siCTL conditions. The distribution is calculated according to the fold change (siFra-1/siCTL) set to ± 1.5.

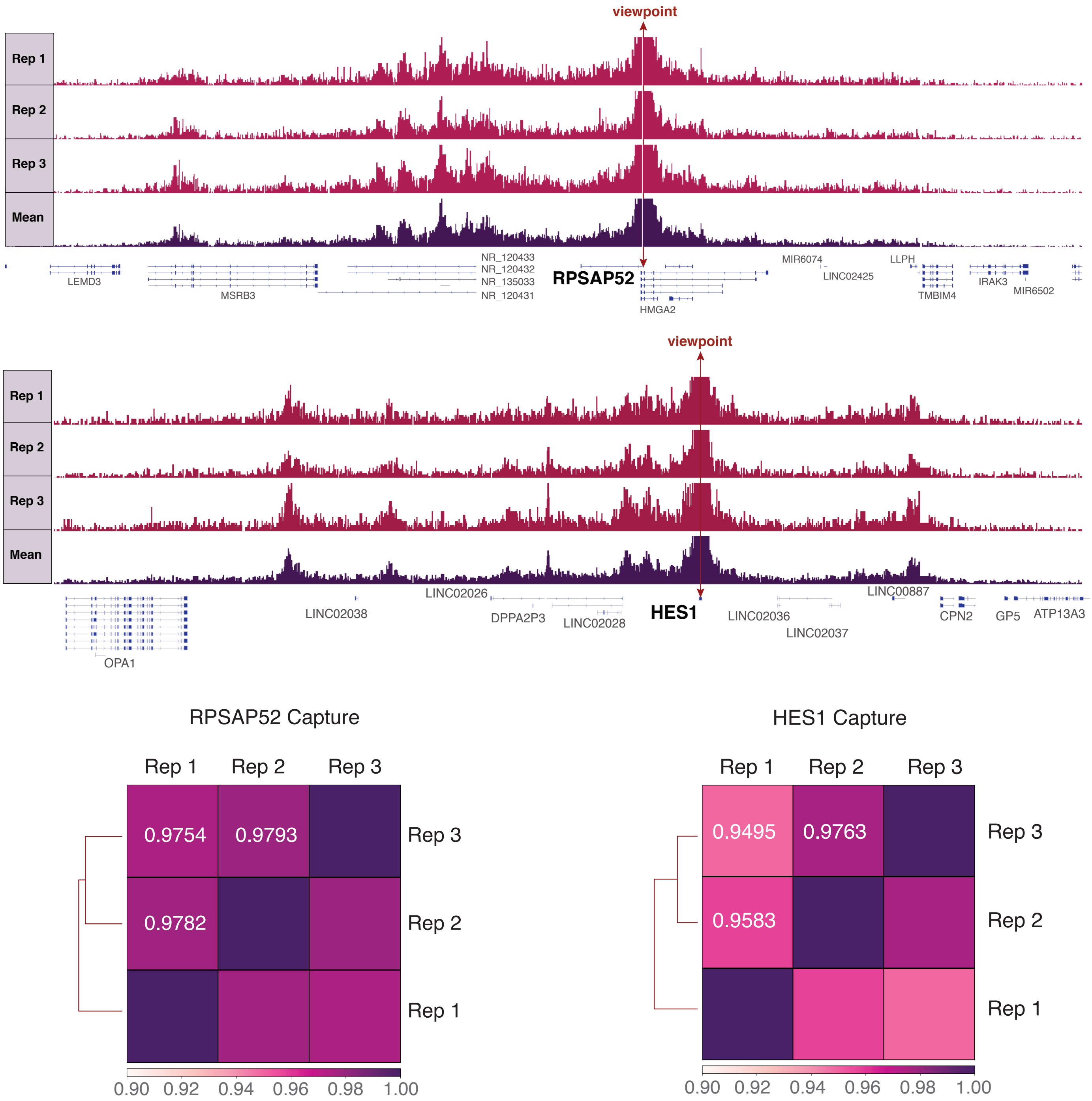

Supplementary Data S9: Biological replicates in NG Capture-C experiments are reproducible. The *RPSAP52* and *HES1* gene loci are shown to illustrate the quality of NG Capture-C data. The three biological replicates, as well as their means, are presented for each gene. Heatmaps in the lower panel represent the Pearson Correlation coefficient of the three biological replicates of each of the two loci in the control siCTL condition.

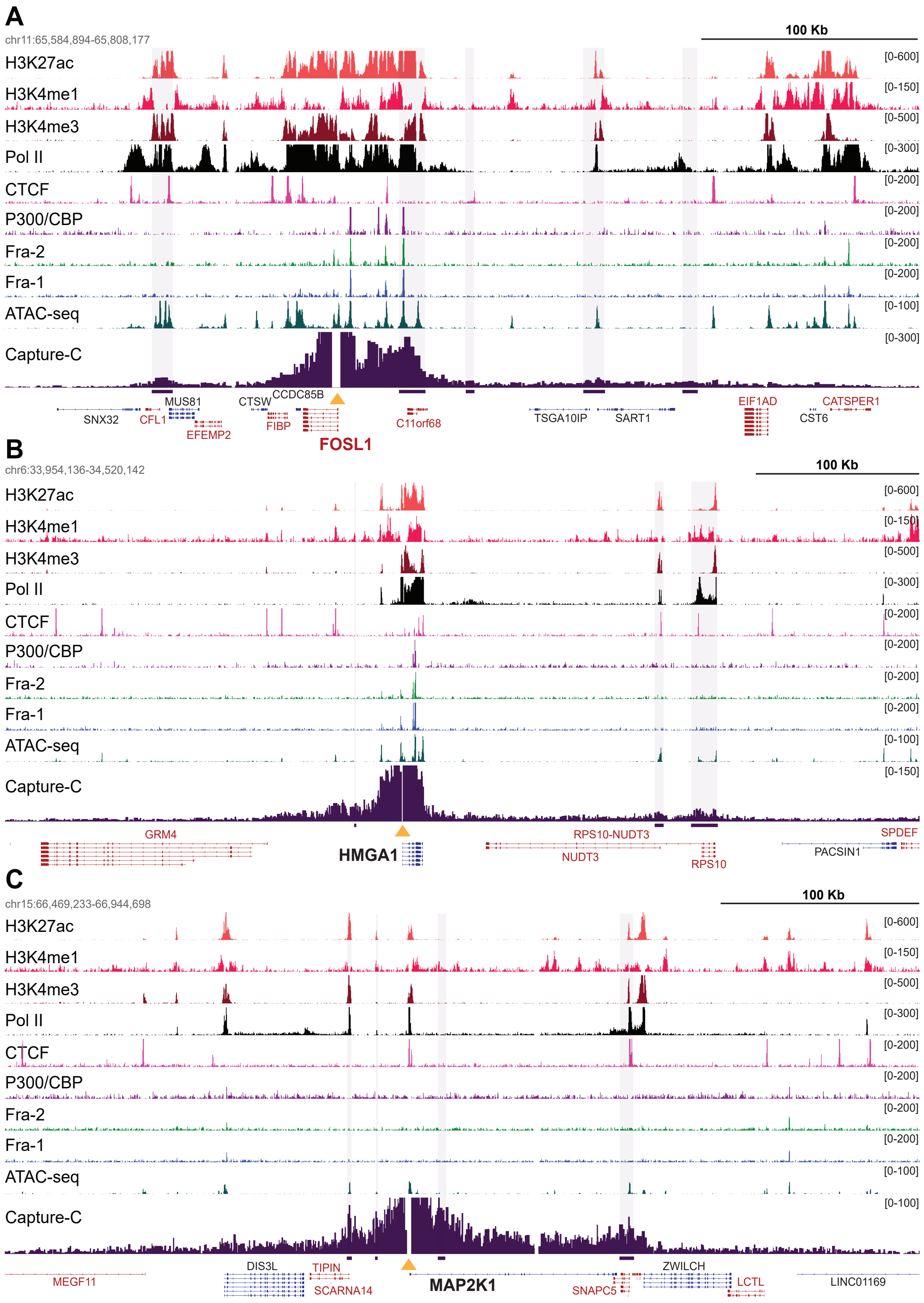

Supplementary Data S10: NG-Capture C profiles obtained for the *FOSL1*, *HMGA1* and *MAP2K1* gene loci. For Capture C, the y-axis represents the normalized number of unique interactions per restriction fragment. PIRs identified using the PeakC R package are highlighted in light purple vertical lines. The scale (100 kb) is indicated at the top right edge of each figure. The yellow triangles indicate viewpoints. Genes encoded by the forward strand are shown in black and those encoded by the reverse strand are shown in red.

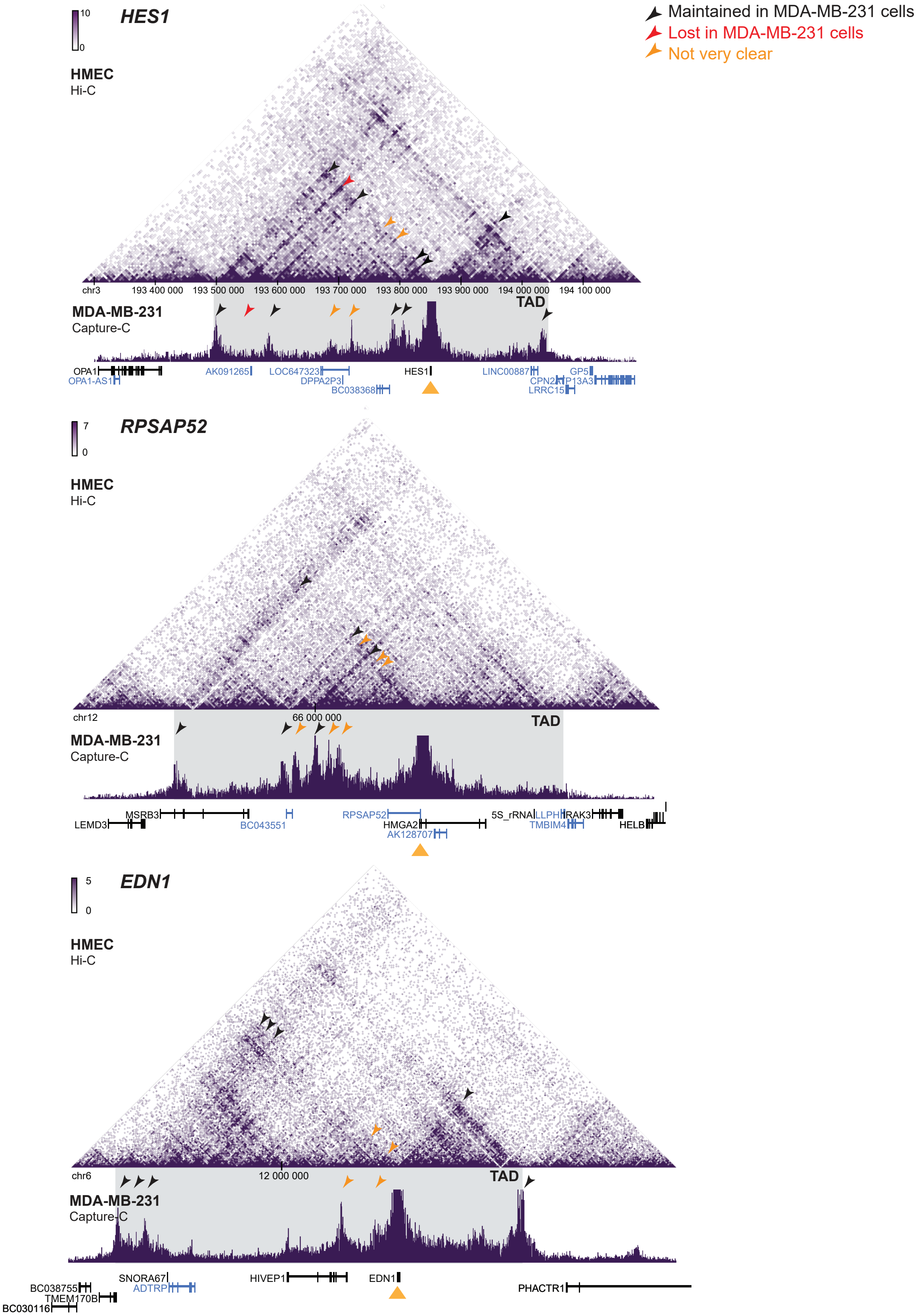

Supplementary Data S11: ***HES1, RPSAP52*** **and** ***EDN1*** **PIRs in the MDA-MB-231 cell line lie within the same TADs as their cognate genes.** The Hi-C contact matrices used were established in primary mammary epithelial cells (HMECs) by Rao et al. (Cell, 2014, 159, 1665-1680). Data were visualized using the 3D Genome Browser described at http://promoter.bx.psu.edu/hi-c/ on the genomic regions encompassing the *HES1*, *RPSAP52* and *EDN1* PIRs identified by NG Capture-C in MDA-MB-231 cells. The grey rectangles separating Hi-C and NG Capture-C data delineate the Topologically-associating domains (TADs) identified in HMECs. The small inclined arrows indicate the interactions that are maintained (black), lost (red) in MDA-MB-231 cells as compared to HMECs or not very clearly identified in the Hi-C map (orange). The yellow triangle below NG Capture-C data indicate viewpoints in these experiments.

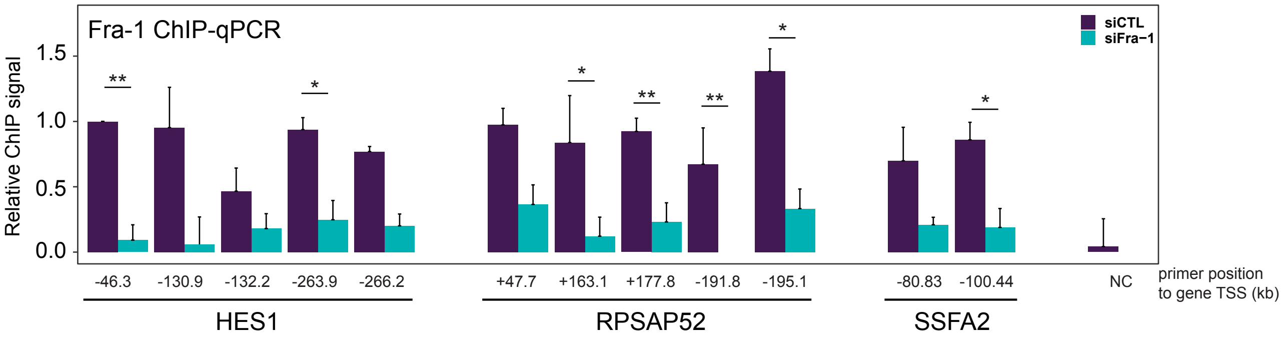

Supplementary Data S12: Fra-1 depletion at the chromatin level upon siFra-1 transfection. Fra-1 binding was analyzed by ChIP-qPCR in the presence and the absence of siFra-1 (siCTL in purple, siFra-1 in blue) at Fra-1-bound PIRs identified by NG Capture-C for 3 genes (HES1, RPSAP52 and SSFA2). Sequences of primers used for qPCR amplifications are given in Supplementary Data S1E. Signals were normalized to input and to the -46.3 signal of the HES1 gene arbitrarily set to 1. Background signal from a non-specific antibody was subtracted. A region devoid of Fra-1 binding was used as negative control (NC). p-values were calculated at each position with respect to the siCTL condition using a two-tailed unpaired t-test. (*) and (**) correspond to p-values < 0.05 and <0.01, respectively (n=3).
